# A flexible hierarchical framework for improving inference in area-referenced environmental health studies 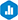


**DOI:** 10.1002/bimj.201900241

**Published:** 2020-06-22

**Authors:** Monica Pirani, Alexina J. Mason, Anna L. Hansell, Sylvia Richardson, Marta Blangiardo

**Affiliations:** 1 MRC Centre for Environment and Health, Department of Epidemiology and Biostatistics, Imperial College London, London, UK; 2 Department of Health Services Research and Policy, London School of Hygiene and Tropical Medicine, London, UK; 3 Centre for Environmental Health and Sustainability, University of Leicester, Leicester, UK; 4 MRC Biostatistics Unit, Cambridge Institute of Public Health, University of Cambridge, Cambridge, UK

**Keywords:** area-referenced studies, Bayesian inference, data integration, missing data, uncertainty

## Abstract

Study designs where data have been aggregated by geographical areas are popular in environmental epidemiology. These studies are commonly based on administrative databases and, providing a complete spatial coverage, are particularly appealing to make inference on the entire population. However, the resulting estimates are often biased and difficult to interpret due to unmeasured confounders, which typically are not available from routinely collected data. We propose a framework to improve inference drawn from such studies exploiting information derived from individual-level survey data. The latter are summarized in an area-level scalar score by mimicking at ecological level the well-known propensity score methodology. The literature on propensity score for confounding adjustment is mainly based on individual-level studies and assumes a binary exposure variable. Here, we generalize its use to cope with area-referenced studies characterized by a continuous exposure. Our approach is based upon Bayesian hierarchical structures specified into a two-stage design: (i) geolocated individual-level data from survey samples are up-scaled at ecological level, then the latter are used to estimate a generalized *ecological propensity score* (EPS) in the in-sample areas; (ii) the generalized EPS is imputed in the out-ofsample areas under different assumptions about the missingness mechanisms, then it is included into the ecological regression, linking the exposure of interest to the health outcome. This delivers area-level risk estimates, which allow a fuller adjustment for confounding than traditional areal studies. The methodology is illustrated by using simulations and a case study investigating the risk of lung cancer mortality associated with nitrogen dioxide in England (UK).

## 1 Introduction

Area-referenced data are collected in a wide range of scientific fields, and among others, are often used in epidemiology and public health research. They refer to a specific ecological study design where data are aggregated within well-defined nonoverlapping spatial boundaries. In environmental health-effect studies, used to assess the health effects of environmental threats (e.g., air pollution, noise, soil contaminants), this kind of study design is extremely popular, as it takes advantage of a complete spatial coverage provided by administrative and registry data.

Despite being representative of the entire population and providing the statistical power for studying risks for even rare diseases, ecological studies can suffer from a number of methodological issues leading to biases (e.g., Greenland, 2001; Greenland & Morgenstern, 1989; Richardson & Monfort, 2000; Richardson, Stücker, & Hémon, 1987; Wakefield & Lyons, 2010; Wakefield & Salway, 2001), which cause a mismatch between conclusions drawn from aggregate data with conclusions that would be obtained from the individual-level data. To face the problem, approaches have been proposed, which integrate ecological-level data with individual-level data from surveys, cohorts, or case-control studies (Haneuse & Bartell, 2011). The scientific interest of these integrative approaches lies typically in individual-level associations and assumes that exposure and/or the outcome of interest are/is available from the individual-level–based studies. However, this is not always the case, for example, for adverse health outcomes such as cancer mortality, individuals data usually do not have full information available on risk factors and personal exposure. In this instance, ecological studies can still represent a useful instrument to face knowledge gaps when it is not feasible to access and process individual-level information on health outcome, exposure, and potential confounders.

In this paper, we propose a methodology in the spirit of the above integrative approach that incorporate information from all levels, but we draw the inferential interest in ecological-level associations. Area-referenced health data are typically obtained from administrative registries (e.g., incidence, mortality or hospital admissions), hence they have limited information on confounding variables. We use external data, available from individual-level survey samples, to supply the lack of information on confounding factors in the routinely collected administrative data sets. The confounding adjustment is resolved at ecological scale by taking advantage of the propensity score methodology, which refers to a class of methods that are used to alleviate the bias in estimating the exposure effect, reducing the likelihood of confounding in analyzing observational data (e.g., Austin, 2011). Propensity score was originally defined by Rosenbaum and Rubin (1983) as the conditional probability of being exposed (or treated) given a set of observed covariates, where the exposure is a binary variable. McCandless, Richardson, and Best (2012) revisited the methodology proposing a conditional propensity score as a tool for integrating data collected in the main individual-level study with data from an external validation data set. Wang, Pirani, Hansell, Richardson, and Blangiardo (2019) extended this idea in the context of small-area regression adjustment, proposing an ecological propensity score, henceforth named EPS, estimated for spatial areas upon external individual-level data obtained from surveys. In lieu of recent contributions such as Zigler et al. (2013) advocating a separation between propensity score estimation and outcome regression analysis for exposure effect estimation, Wang et al. (2019) proposed a two-stage approach, where first the EPS is estimated upon individual-level data for the areas covered by a survey (in-sample areas), then it is imputed in the out-of-sample areas and included in the health-effect ecological regression model for confounding adjustment. In line with the predominant literature on propensity score adjustment, the work of Wang et al. (2019) is, however, limited to a setting where the exposure is binary. Moreover, uncertainty propagation between stages is not well exploited and sparsity in the geographical coverage of the individual-level data is by default assumed to comply with the mechanism of missing at random (MAR; Rubin, 1976).

The approach presented in this paper extends and generalizes the work by Wang et al. (2019) to deal with unmeasured confounding adjustment in area-referenced studies. The major contributions are threefold. First, we provide a generalization of the EPS to cope with a more realistic situation in environmental health-effect studies where the exposure is a continuous variable and we call it a *generalized EPS*. Literature studying generalized versions of propensity score is exclusively framed within inference for individual-level–based epidemiological studies (Hirano & Imbens, 2004; Imai & van Dyk, 2004; Imbens, 2000) and there is still a lack of contributions on exploiting the potential benefits in using this methodology in ecological studies. Second, we address the problem of sparsity in space of the potential confounding factors derived from individual-level survey samples, considering both MAR and missing not at random (MNAR; Rubin, 1976) mechanisms. Third, we address the issue of uncertainty propagation in the adopted two-stage sequential approach, allowing uncertainty in the generalized EPS estimation to feed into the analysis stage.

The effectiveness of our approach is demonstrated through a simulation study and a real application investigating the risk of lung cancer mortality associated with outdoor nitrogen dioxide (NO_2_) exposure in England (UK). Inference is carried out within a Bayesian framework using Markov chain Monte Carlo (MCMC) methods.

The paper is structured as follows. Section 2 describes the applied case study. Section 3 presents a brief review of the area-based modeling background and outlines our approach for unmeasured confounding adjustment. Section 4 describes the simulation study, designed to account for different scenarios of sparsity of the individual-level data. Section 5 presents the application of the approach to the case study. Finally, Section 6 discusses the relevance of our findings including future work.

## 2 Case Study Description: Lung Cancer Mortality and NO_2_ in England

Our work is motivated by a population study investigating the risk of lung cancer mortality among adult residents in England associated with outdoor NO_2_ exposure. NO_2_ is a traffic-related gaseous air pollutant that has been previously associated with lung cancer (e.g., Fischer et al., 2015; Hamra et al., 2015; Vineis et al., 2006). The spatial units on which we built the ecological analysis are the 326 local authority districts (LADs) in England with the geographical boundaries matching the 2011 UK census. In the remaining of this section, we present the different sources of data used for the case study.

### 2.1 Ecological-level data

Mortality data for lung cancer were identified from the International Classification of Disease 10th Revision (ICD-10) codes C33–C34. These data are held by the UK Small Area Health Statistics Unit (SAHSU) at Imperial College London, and supplied by the Office for National Statistics (ONS). Age, gender, and LAD of residence were extracted for each record. After removing 37 cases whose LAD could not be identified, and 28 cases with missing information on age, there were 56,651 deaths from lung cancer in England over the period 2011–2012. Of these, 31,460 were among men and 25,191 among women. The age groups for the resident cases and population aged 15 and over, were organized in five-year age bands up to 84 years, and 85 years and older. Based on the population composition in each LAD, we calculated the expected numbers of cancer deaths adjusting for age and gender using internal standardization. Then, the crude ecological estimation of mortality risk for each LAD is described by the standardized mortality ratio (SMR), that is the ratio between observed and expected cases.

Ambient air pollution concentrations of NO_2_ (μg m^−3^) for the year 2001 were obtained by land use regression (LUR) technique (Hoek et al., 2008) on a grid of 100 m × 100 m resolution. The details of the model are published in Vienneau et al. (2010). LUR is a widely used method for modeling spatial variability in outdoor air pollutants and it has been recommended by the Health Effects Institute (HEI, 2010) as a preferred method for estimating exposure to spatially heterogeneous pollutants such as NO_2_. LUR uses multiple linear regression to examine the associations between measured pollutant concentrations from air quality networks and geographical covariates such as traffic, land use, population density, meteorology, and physical geography. Then, it predicts the air pollution concentrations in areas without air quality measurements using the parameter estimates derived from the regression analysis and taking into account site-specific variables. In the Supporting Information (Section S1.1), we provide more details on the LUR model and the method used to convert the grid format of the data to the vector format (polygon/area) for England required by our analysis.

**Figure 1:**
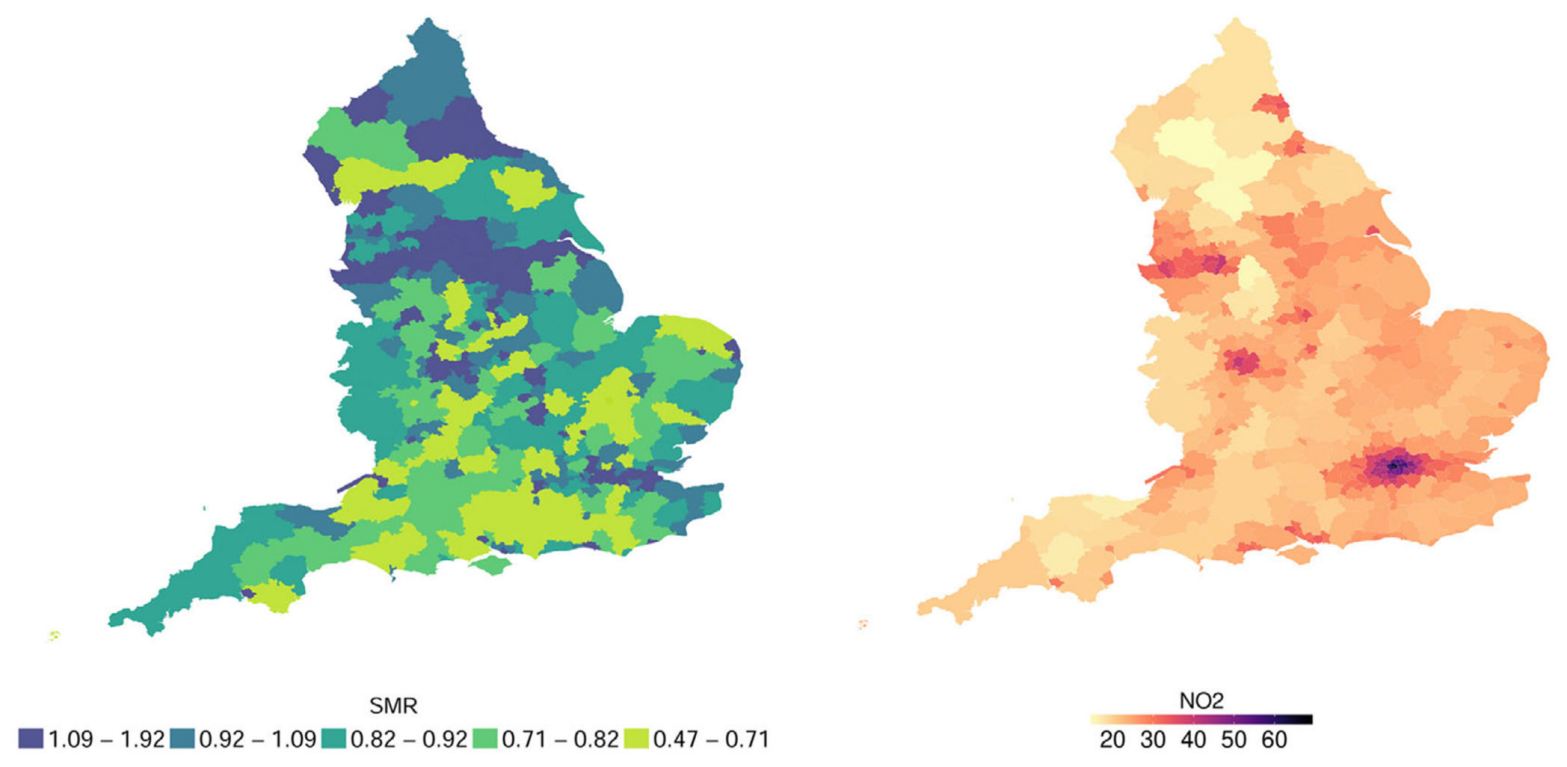
Distribution of SMRs (2011-2012) and NO_2_ (2001) in England

Figure 1 displays the distribution of lung cancer SMRs and NO_2_ concentrations in England, while Figure S2 in the Supporting Information (Section S1.2) presents the plot of their relationship. Note that, for modeling purpose, we use NO_2_ concentrations on the square root scale, as is common practice in modeling air pollutant data (e.g., Lee, Mukhopadhyay, Rushworth, & Sahu, 2017), which are typically nonnegative and skewed to the right, although the effect estimates are given on the original scale. Also note that, as cancer is a disease with a long latency period, it is important to consider a lag between exposure and health occurrences. Based on the work of Pedeli, Hoek, and Katsouyanni (2011), in the present study, we consider on average 10–11 years of latency.

The ecological LAD-level covariates available for our analysis are the Carstairs index and the average of home radon measurements. More specifically, the Carstairs index is used as the LAD measure of deprivation as it has been shown to be a strong predictor of cancer mortality (Jolley, Jarman, & Elliott, 1992). The index is derived from 2001 UK census variables (household overcrowding, male unemployment, car ownership, and head of household in low social class). The LADs are allocated to deprivation quintiles, from least deprived to most deprived.

Moreover, we consider home radon measurements (Bq m^−3^), as radon is the second leading cause of lung cancer (after smoking) in the general population, and it is responsible for around 1,100 lung cancer deaths a year in the United Kingdom (HPA, 2009). It is a naturally occurring radioactive gas that emanates from rocks and soil as a result of radioactive decay of uranium. Radon escapes easily from the ground (or water) into the atmosphere, where its decay produces further radioactive particles. It may be found in outdoor and indoor environments. However, while radon dilutes to very low concentrations in outdoor spaces, not being generally a threat, it can accumulate to high concentrations in indoor spaces (WHO, 2009). We use freely available radon measurements grouped at district level (https://www.gov.uk/government/publications/radon-in-homes-in-england-2016-data-report), represented by the arithmetic average over 525,000 measurements conducted in homes in England in the period between 1980 and 2015. Initial explorative analysis of the radon data showed an increase of the variance in the measurements as the mean increases. To stabilize the variance and encourage normality, we transformed the original measurements of radon on the square root scale.

In the Supporting Information (Section S1.3), we provide the maps of the two ecological-level covariates.

### 2.2 Individual-level data

Measurements at individual-scale are obtained from the Health Survey for England (HSfE), held by SAHSU, which provides information about various aspects of people’s health and behaviors. To comply with the latency period used between NO_2_ exposure and mortality, we consider the 2001 survey. The sample for that year was designed to be a cross section of the population living in private households in England and 13,680 addresses were drawn from the postcode address file (note that at each address all persons were eligible for inclusion in the survey). We included the following survey information: cigarette smoking status (current cigarette smoker, ex-regular cigarette smoker, never regular cigarette smoker), passive smoking (nonsmoker but living with at least one smoker in the household), alcohol consumption (up to weekly limits and over weekly limits), longstanding diseases (infectious diseases, neoplasms, endocrine and metabolic diseases, respiratory diseases), education (national vocational qualification 4 or 5 or degree or equivalent), and ethnicity (white or any other ethnic group). The survey samples on these potential confounders provided a spatial coverage of 263 LADs (80.7%). The median number of subjects for each LAD is 55, with a range spanning from 1 to 351 subjects. Of the 263 LADs, only six had ≤5 subjects with recorded information on the potential confounders.

## 3 A Two-Stage Sequential Bayesian Approach for Improving Inference in Area-Referenced Studies

### 3.1 Notation and background

We begin by briefly describing the modeling background for our approach, based on hierarchical spatial regression for count areal data. Consider a geographical domain *𝒟* as a finite collection of disjoint areal units, indexed by *i* = 1, 2, …, *N*. In the typical area-referenced environmental epidemiological study the data in analysis consist of (i) the disease counts, Y = (*Y*_1_, …, *Y_N_*); (ii) the expected number of cases, **E** = (*E*_1_, …, *E_N_*), based on population size and demographic structure (e.g., age and gender) within area i; (iii) the area-level exposure of primary interest, **X** = (*X*_1_, …, *X*_N_); and (iv) a collection of Q measured area-level confounding variables, denoted by an *N* × *Q* matrix **C**_*i*_. Then, the commonly used Bayesian hierarchical formulation for a (rare) health response variable with support ***S*** = {0, 1, 2, …}, which we call *the naïve ecological model*, is given by:

(1) $$\begin{array}{l} \;Y_{i}|\mu_{i}∼\text{Poisson}\left(E_{i}\mu_{i}\right),\;i,\ldots ,N,\\ \;\text{log}(\mu_{i})=\beta_{0}+X_{i}\beta_{X}+{\text{C}}_{i}^{⊤}\beta_{C}+\theta_{i}+\phi_{i},\\ \;\theta_{i}|\sigma_{\theta }^{2}∼\text{N}(0,\sigma_{\theta }^{2}),\\ \phi_{i}|\phi_{s},\sigma_{\phi }^{2}∼\text{N}\left(\frac{1}{n_{i}}\underset{s\in \partial_{i}}{\sum^{\;}}\phi_{s},\frac{\sigma_{\phi }^{2}}{n_{i}}\right),\;i\neq s, \end{array}$$

where *μ_i_* is the mean parameter and represents the unknown log-relative risk of disease (or mortality), ß_0_ is the intercept that quantifies the logarithm of the global risk, *β_X_* is the coefficient quantifying the health effect for the exposure of interest, and ***β**_c_* is a vector of *Q* × 1 parameter coefficients associated with the area-level covariates (note that in (1) the covariates are specified to have a linear relationship with the outcome, but a more flexible parameterization can be set up). The *N*-length parameter ***θ*** = (*θ_i_* …, *θ_N_*) specifies exchangeable random effects, capturing heterogeneity among the areas, while *ϕ* = (*ϕ_i_*…, *ϕ_N_*) accounts for a residual spatial dependence and is typically described by models based on Markov random fields (MRFs), such as the auto-Gaussian models, popularly called conditional autoregressive (CAR) models (Besag, 1974; Besag, York, & Mollié, 1991). In (1) ***ϕ*** is specified by an intrinsic CAR (ICAR), where *s* ∈ *∂_i_* represents the set of neighbors of the *i*-th area and *n_i_* denotes the number of neighbors, while $\sigma_{\phi }^{2}$ is the conditional variance parameter that controls the amount of variation between the random effects (i.e., variance decreases as the number of neighbors increases). Here, impropriety in the CAR prior is resolved by constraining the spatial random effects to sum to zero. The inclusion of both the residual terms ***θ*** and ***ϕ***, is known as a convolution model (Besag et al., 1991). We note, however, that alternative specifications of the ICAR prior (e.g., Leroux, Lei, & Breslow, 2000) and extensions to capture localized autocorrelation (e.g., Lee, Rushworth, & Sahu, 2014) have been also proposed, but here we refer to the Besag et al. (1991) specification as easily implemented in largely used softwares such as BUGS and Stan.

Now, suppose that, within the same geographical domain, we have data from a representative sample of subjects directly measured on *K* variables collected within individual-based studies, which are potential confounders of the association between health outcome and exposure of interest. Our starting point is that, if we are able to spatially locate these subjects within the area units in ***𝒟***, we can up-scale the individual-level data to ecological scale to form latent confounding factors that we could not measure directly at ecological level. Because individual-level data are usually sparse on ***𝒟*** (i.e., they may be missing in space), we are not able to provide a complete geographical coverage of the latent confounders. Therefore, we summarize the information held by these latent factors for the insample areas in the generalized EPS. In this way, just a single score needs to be imputed in the areas where there is no individual-level information, largely simplifying both statistical analysis and computation. Then, the estimated and imputed generalized EPS is included as an additional term in the health-effect regression model for confounding adjustment.

Overall, the proposed modeling strategy results in a Bayesian two-stage sequential approach. In the first stage, that we call the *design stage*, we jointly up-scale a set of measured spatially located individual-level confounders to the ecological level, and estimate the generalized EPS for the in-sample areas. In the second stage, called the *analysis stage*, we implement a health-effect regression model. A two-stage approach is practical from a computational perspective but has also been supported by previous studies (e.g., Zigler, 2016; Zigler et al., 2013), which discuss how the feedback of information between the two stages could lead to biased estimates of the health effect. However, a disadvantage associated with Bayesian two-stage approaches is that uncertainty in the estimated quantities in the first stage is not carried forward to the second stage. In Section 3.4, we show how to overcome this issue providing information from the design stage into the analysis stage for the in-sample areas.

### 3.2 Design stage: individual-level data up-scaling

Let **M**_*i*_ = (*M*_*i*1_, …, *M*_*iK*_) denoteasetof *K* potential confounders at the area level (*i* = 1, …, *N*; *k* = 1, …, *K*), which we need for the ecological inference. The **M***_i_* are *unmeasured*, however on *j* = 1, …, *J_i_* individuals we have measurements on the same *K* potential confounders, denoted by **m**_*ij*_ = (*m*_*ij*1_,…, *_mijk_*), from a sample survey covering a subset of areas, that is, *i* = 1, …, *S* ∈ *N*.

The observed individual-level data **m**_*ij*_: (i) may be mixed types (e.g., binary, categorical, or continuous data), (ii) may be characterized by different degrees of correlation among them, and (iii) may hold a spatial structure. Thus, we assume that the individual-level variables belong to a distribution from the exponential family, where the expectation *M_ik_* is linked to predictors through a suitable link function *g_k_*(), whose choice determines the type of model being fit. Specifically, if the *k*-th variable is (i) binary with support ***S*** = {0,1}, we model *m_ij_* ~ Bernoulli(*M_i_*) (where *M_i_* is the probability), with logit or probit link function; (ii) categorical with support *S* = {1,…, *T*} (i.e., it can take on one of *T* possible categories), we model *m_ij_* ~ Categorical(***M**_i_*), where the vector ***M**_i_* contains the probabilities of the variable for each of the categories (the sum of *M_i_* is 1), with logit or probit link function; (iii) continuous with support *S* = {−∞, ∞}, we model $m_{ij}∼\text{N}(M_{i},\sigma_{k}^{2})$, with identity link function. Then, we assume that *g_k_*(*M_ik_*) is a function of a variable-specific intercept *ν_k_* (note that, in the case of a categorical variable, we have more that one intercept term, equal to the number of categories minus 1 to ensure identification), and *S* × *K* spatially dependent random effects. The up-scaling of the individual-level variables is achieved as follows:

(2) $$\begin{array}{l} g_{k}\left(M_{ik}\right)=v_{k}+\xi_{ik},\;i=1,\ldots ,S,\;k=1,\ldots ,K,\\ \xi_{i}|\xi_{s},\Sigma_{\xi }∼\text{N}\left(\frac{1}{n_{i}}\underset{s\in \partial_{i}}{\sum^{\;}}\xi_{s},\frac{1}{n_{i}}\Sigma_{\xi }\right),\;i\neq s. \end{array}$$

In (2), ***ξ***^⊤^ = (***ξ***_1_, …,***ξ**_S_*), where each ***ξ**_i_* = (*ξ_i1_*…, *ξ_iK_*)^⊤^, are spatial random effects modeled with multivariate MRF for lattice data, offering flexibility in modeling the spatial cross-dependencies between these variables. Here, we consider a multivariate intrinsic conditionally autoregressive (MICAR) prior (Mardia, 1988), a special case of MRF where the conditional distributions are assumed to be Gaussian. This prior allows us to model spatial dependence across the geographical areal units while accommodating the correlation structure among the *K* individual-scale variables. In the MICAR representation in (2) expressed via full conditional distributions, the symbol specifications are as in (1). Because the *K* individual-level variables from external data sets such as surveys do not have complete spatial coverage, we adopt a distance-based neighborhood structure (distances are measured between area centroids), ensuring that each area has at least one neighbor. The improperty in the MICAR model is resolved by applying a sum to zero constraint to (***ξ***_1_,…, ***ξ**_S_*). The MICAR specification implies that the covariances between the individual-scale variables are invariant across the areas (i.e., a separable dispersion structure is assumed). Less restrictive specifications have been advanced in literature (e.g., Jin, Banerjee, & Carlin, 2007), however they lead to more complex dispersion structures.

We define a Wishart distribution prior for the *K* × *K* nonsingular precision matrix, that is, $\Sigma_{\xi }^{-1}|v$, **B** ~ Wishart_*K*_(*v*, ***B***), where *v* are the degree of freedoms, set to *v* > *K* – 1 in order for the distribution to be proper and **B** is the *K* × *K* positive definite scale matrix with a prior equal to the inverse of the correlation matrix between the individual-level variables. Finally, the *K* intercepts follow improper uniform prior distributions (from −∞ to −∞), that is, *ν_k_* ~ flat(), to ensure that the join prior distribution for the intercepts and the constrained MICAR random effects is equivalent to un intrinsic MICAR prior on the unconstrained random effects (Lunn, Jackson, Best, Thomas, & Spiegelhalter, 2013).

### 3.3 Design stage: generalized EPS estimation

The multivariate spatial generalized linear mixed model in (2), is jointly modeled with the generalized EPS estimation, so that the uncertainty from the former feeds into the latter, and areas with large survey samples would be characterized by lower variability than those from a small sample size. We assume here that the exposure of interest is continuous, with support **X** ⊂ ***R*** and **X** ∈ *χ*, where *χ* is the sample space of the exposure variable. Within observational individual-level study designs, Hirano and Imbens (2004) and Imai and van Dyk (2004) define a generalized version of a standard propensity score for a continuous exposure as the conditional probability density function of the exposure given the observed confounders, that is, ***P_δ_*** = **X|*C_i_***, where *δ* parameterizes the distribution, estimated via Gaussian regression of exposure on confounders. Then, Hirano and Imbens (2004) and Imai and van Dyk (2004) demonstrated that a generalized propensity score for a continuous exposure hold the same key properties derived originally by Rosenbaum and Rubin (1983) for the standard propensity score. Here, we define the generalized EPS conditional on observed confounders, similarly to McCandless et al. (2012) and Wang et al. (2019), and estimated for the in-sample area $i\in 1,\ldots ,S\;\text{as}\;Z_{i}^{\left(0\right)}$. In particular, given the area-level observed potential confounders **C**_*i*_ and the latent confounders **M**_*i*_ obtained through the up-scaling of the individual-level variables in (2), the generalized EPS for the in-sample areas, that is, $Z_{i}^{\left(0\right)}$, is obtained from the following Gaussian conditional distribution for the exposure:

(3) $$\begin{array}{l} X_{i}|{\text{C}}_{i},{\text{M}}_{i}∼\text{N}(\delta_{0}+{\text{C}}_{i}^{⊤}\delta_{C}+{\text{M}}_{i}^{⊤}\delta_{M},\sigma_{X}^{2}),\;i=1,\ldots ,S,\\ \text{where}\;{\text{M}}_{i}^{⊤}\delta_{M}\equiv Z_{i}^{\left(0\right)}\text{.} \end{array}$$

In (3), $Z_{i}^{\left(0\right)}$ is the conditional generalized EPS for the in-sample areas, as the linear combination of the up-scaled individual-level variables and the associated coefficients estimated for **X**, conditionally on the observed ecological-level covariates. Weakly informative priors are chosen for the model parameters, such that **δ** = (*δ*_0_, *δ*_c_, *δ*_M_) ~ N(0,100) and $\sigma_{X}^{-2}$ ~ Gamma(1, 0.1). McCandless et al. (2012) proved the properties of the score constructed in this fashion with respect to a binary exposure. From their results, it follows that the generalized EPS, estimated in (3), holds a balancing property, conditional on the observed potential confounders **C**_i_. In fact, it balances the distribution of the unmeasured confounders **M**_i_ with respect to the exposure *X_i_*, conditional on **C**_i_. Then, we have that for the same value of $Z_{i}^{\left(0\right)}$ conditional on **C**_i_, the probability that *X_i_*, = *x_i_*, does not depend on the value of **M**_i_, that is, **M**_i_ ⫫ 1{*X_i_*, = *x_i_*{|**C**_*i*_, $Z_{i}^{\left(0\right)}$, where **1** is the indicator function. Also, McCandless et al. (2012) show that $Z_{i}^{\left(0\right)}$, conditional on **C**_*i*_, contains all the information about the distribution of *X_i_*, given **M**_*i*_,: **P**(*X_i_*, = *x_i_*|**C**_*i*_, **M**_*i*_) = **P**(*X_i_* = *x_i_*|**C**_*i*_, $Z_{i}^{\left(0\right)}$). Consequently, a key point is that in the analysis stage, to control for the residual confounding captured by **M**_*i*_,, we can estimate the effect of the exposure on the health outcome by modeling the conditional distribution of *Y_i_*, given (*X_i_*, **C**_*i*_, $Z_{i}^{\left(0\right)}$).

### 3.4 Analysis stage: health-effect assessment

The second stage of our modeling framework consists in linking the exposure of the interest to the health outcome accounting not only for the observed ecological confounders but also for the additional confounders estimated through the generalized EPS.

As previously specified, we prevent the feedback between the design stage, where the generalized EPS is estimated and the analysis stage, where the outcome is regressed on the exposure, after including the confounders. Nevertheless, we allow propagation of uncertainty from the design stage to the analysis stage by embedding the latter with an *uncertainty model* for the generalized EPS estimated for the in-sample areas. In particular, let us define the missing generalized EPS for the out-of-sample areas as **Z**^(1)^, such that **Z** = (**Z**^(0)^, **Z**^(1)^). We assume that the generalized EPS estimated for the in-sample areas, **Z**^(0)^, can be treated as an unknown quantity, called **Z**^(*0)^. We write **P**(**Z**^(*0)^ |**λ**) to represent the conditional density function over **Z**^(*0)^, parameterized by λ. Then, we assume a Gaussian distribution letting $\lambda =({\hat{\text{Z}}}^{\left(0\right)},{\hat{\sigma }}_{{\text{Z}}^{\left(0\right)}}^{2})$, which are, respectively, the posterior mean and variance of the generalized EPS estimated in the design stage for the in-sample areas.

Then, because the score is estimated in (3) only for the in-sample areas, we define the *imputation model* for the generalized EPS in the out-of-sample areas, which requires the specification of the assumption regarding the missingness mechanism for the generalized EPS. This is defined via a joint probability distribution for the estimated and missing generalized EPS. The two possible types of mechanisms we consider are MAR and MNAR. Note that missing completely at random (MCAR) is a special case of MAR and it is generally unlikely to occur. In a Bayesian perspective, this requires building an imputation model to predict the missing values, where the generalized EPS is treated as a random variable rather than a fixed covariate (Mason, Richardson, Plewis, & Best, 2012).

#### 3.4.1 Analysis stage under the assumption of a MAR mechanism for the generalized EPS

Under an MAR mechanism, the probability of the generalized EPS being missing in area *i* does not depend on the values of the generalized EPS, after controlling for the other measured factors. In this specific case, the analysis stage of our approach is structured as follows:

*Health outcome model*

(4a) $$\begin{array}{l} \;Y_{i}∼\text{Poisson}\;\left(E_{i}\mu_{i}\right),\;i=1,\ldots ,N,\\ \text{log}\left(\mu_{i}\right)=\beta_{0}+X_{i}\beta_{X}+{\text{C}}_{i}^{⊤}\beta_{C}+f\left(Z_{i}\right)+\theta_{i},\\ \;\theta_{i}|\sigma_{\theta }^{2}∼\text{N}(0,\sigma_{\theta }^{2}), \end{array}$$

where

$$Z_{i}=\{\begin{array}{c} Z_{i}^{\left(*0\right)}\;\text{if}\;i\in S\\ Z_{i}^{\left(1\right)}\;\text{if}\;i\notin S \end{array}$$

with *uncertainty model* for the generalized EPS for the in-sample areas

(4b) $$Z_{i}^{\left(*0\right)}∼\text{N}({\hat{Z}}_{i}^{\left(0\right)},{\hat{\sigma }}_{Z^{\left(0\right)}}^{2}),$$

and *imputation model* for the generalized EPS under MAR mechanism

(4c) $$\begin{array}{c} Z_{i}|X_{i},{\text{C}}_{i},\varphi_{i}∼\text{N}(\gamma_{0}+X_{i}\gamma_{X}+s_{q}({\text{C}}_{i})+\varphi_{i},\sigma_{Z}^{2}),\\ \varphi_{i}|\varphi_{s},\sigma_{\varphi }^{2}∼\text{N}\left(\frac{1}{n_{i}}\underset{s\in \partial_{i}}{\sum^{\;}}\varphi_{s},\frac{\sigma_{\varphi }^{2}}{n_{i}}\right),\;i\neq s. \end{array}$$

In (4a), the parameter interpretation remains unchanged from (1). Here, an additional term of spatial random effects **φ** can be included in the presence of residual spatial dependencies, as specified in (1). Weakly informative priors are chosen for the parameters *β*_0_, *β_X_*, and ***β**_C_* with distribution N(0, 10). Note that the linear relationship between the health outcome and the observed ecological-level covariates **C**_*i*_ can be relaxed using flexible functions. The relationship between the health outcome *Y_i_* and the generalized EPS *Z_i_* is typically not obvious and might be nonlinear. We recommend that, based on the in-sample areas, the functional form that better depicts the shape of this relationship is chosen. In the simulation study, we show the effect of a misspecified *f*() on the health-effect model, comparing linear and polynomial representations, when the relationship between *Y_i_* and *Z_i_* may assume a quadratic form. In the case study, we deal with nonlinearity using spline functions (see Section 5.1).

The estimated value of the generalized EPS to be assigned to areal unit *i* for confounding adjustment is evaluated by using indicator functions where the “if statements” are computationally implemented via step functions. In (4b), the values of the generalized EPS for the in-sample areas are estimated from the uncertainty model. In (4c), the imputed values for the generalized EPS depend on the posterior mean parameter of the conditional model for the generalized EPS estimated in the analysis stage (given by ${\hat{Z}}_{i}^{\left(0\right)}$), the observed ecological variables **X** and **C**_*i*_, as well as on the feedback from the outcome model about the relationship between **Y** and **Z** (adjusted for **X** and **C**_*i*_). Here, the shape of the relationship between the generalized EPS and the observed ecological-level covariates **C**_*i*_ is allowed to be flexible and data driven (Wang et al., 2019), so that the functions *s_q_*() can be used to relax the assumption of linearity. The imputation model can include the spatially structured component *φ* as the generalized EPS, estimated in (3) for the in-sample areas *S*, may inherit spatial structure from the individual-level variables **m**_*ij*_ upon which it is constructed. In (4c) it is modeled through an ICAR specification, implying that areas closest in terms of distance behave similarly with respect to the generalized EPS imputation distributions. For the variance parameter of this prior, $\sigma_{\varphi }^{2}$, we assume an inverse-Gamma distribution, with hyperparameters equal to 2 and 1, respectively, based on Lee and Sahu (2016), making the prior proper (such that proper posteriors ensue for each of the *φ_i_*) but weakly informative.

#### 3.4.2 Analysis stage under the assumption of an MNAR mechanism for the generalized EPS

Under an MNAR mechanism, the probability of the generalized EPS being missing in area *i* depends on the missing values of the generalized EPS themselves, after controlling for the other measured confounders. In this scenario, we need to model the missingness mechanism through a missing indicator variable for the generalized EPS (Mason et al., 2012).

We introduce a binary indicator variable, *ℓ_i_*, ∈ {0,1}, indicating whether the individual-scale variables and therefore *Z_i_*, are/is available or missing in area *i*. Under an MNAR mechanism, the generalized EPS and the probability of missingness have a joint distribution that can be specified in different ways, depending on how the joint distribution is factorized. We use a selection model (Molenberghs, Fitzmaurice, Kenward, Tsiastis, & Verbeke, 2015), which specifies a joint distribution of *ℓ* and **Z**, through models for the marginal distribution of **Z** and the conditional distribution of *ℓ* given **Z**, that is, **P**(**Z**, *ℓ*|**Ω**,**Λ**) = **P**(**Z**|**Ω**)**P**(*Λ*|**Λ**, **Z**), where **Ω** is a set of parameters that describes the distribution of **Z** and **Λ** is a set of parameters that describes the missingness function. Thus, keeping (4a), (4b), and (4c) unchanged, we add a new submodel in the analysis stage, that we term the *missingness model*, that is,

(5) $$\begin{array}{c} \ell_{i}∼\text{Bernoulli}(\pi_{i}),\\ \text{logit}\left(\pi_{i}\right)=a_{0}+X_{i}a_{X}+{\text{C}}_{i}^{⊤}{\text{a}}_{C}+Z_{i}a_{Z}. \end{array}$$

We specify mildly informative priors (i.e., N(0,10)) for the parameters of the missingness model to encourage identifiability (Mason et al., 2012). Moreover, because the estimation of the parameter *a_Z_* relies on the assumption about its functional form and due to the difficulty associated with the estimation (as the parameter is unidentifiable from the data alone), we performed several sensitivity analyses fixing it to different values. We describe these analyses, carried out using simulative examples, in the Supporting Information (Section S2.1).

**Figure 2:**
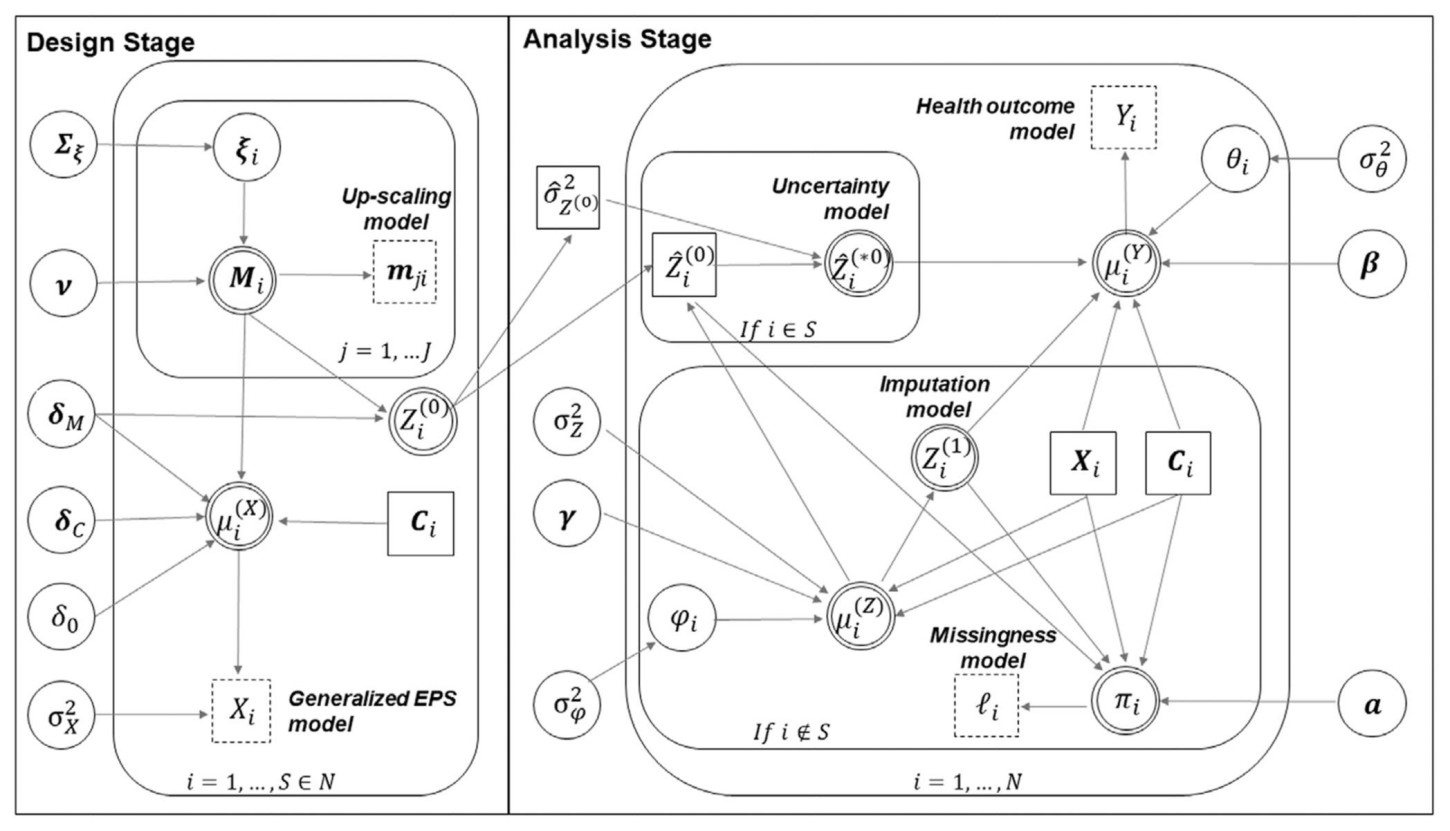
Graphical representation of the two-stage sequential Bayesian approach. Each quantity corresponds to a node and links between nodes show direct dependence. The notation is defined as follows. Squares denote data, distinguished in unmodeled (solid line) or modeled (dashed line). Circles (solid line) represent stochastic parameters, while circles with a double line indicate logical nodes (i.e., parameters that are defined as a function of other random quantities). Repetitive structures, such as loops (e.g., *i* = 1, …, *N*), are represented by plates. Note that node $\mu_{i}^{\left(X\right)}$ in the design stage is the mean of the regression model for **X** in (3). In the analysis stage, $\mu_{i}^{\left(Y\right)}$ is the log relative risk in the health outcome model in (4a), while $\mu_{i}^{\left(Z\right)}$ is the mean of the imputation model for **Z** in (4c). A caveat due to the use of WinBUGS in implementing our approach, is that we cannot use the latent **Z**^(*0)^ as an estimate of the generalized EPS from the design stage in the imputation model within the analysis stage, as this would incur a multiple specification of the same stochastic node. Therefore, for the specification of the generalized EPS for the in-sample areas within the imputation model, we set $Z_{i}^{\left(0\right)}={\hat{Z}}_{i}^{\left(0\right)}$

Figure 2 depicts the graphical form of our two-stage modeling approach. Note that as the design and analysis stages are fitted separately, we prevent the feedback between the latter to the former, which would influence the estimate of Z^(0)^. In this, our analysis differs from recent contributions on propensity score methodology under Bayesian inference (e.g., An, 2010; McCandless et al., 2012), which instead proposed a single procedure in the fashion of a Bayesian full probability model. Finally, regarding the analysis stage, we would like to underline that, because uncertainty, imputation, and health outcome models are fitted jointly, information flows across submodels.

### 3.5 Computation

We use the R software (R Core Team, 2019) for data processing, summarization, and plotting, and the open-source WinBUGS software (Lunn et al., 2013) for making inference on the model parameters through MCMC. The number of samples, thinning, and burn-in changed according to the convergence requirements for the simulation study and the case study. In particular, for the latter, we run two parallel sampling chains of length 100,000. We discarded the first 80,000 samples as burn-in and among the remaining 20,000 we retained one every 10 iterations for posterior inference. To diagnose convergence, we used the CODA package in R, monitoring the chain mixing, autocorrelations, and densities, and checking the Gelman–Rubin diagnostics (Gelman & Rubin, 1992). Due to confidentiality, the case study’s models were run on a private network, which is an isolated air-gapped network not connected to any other network with a mandatory access control system. Using an Intel(R) Xeon(R) CPU E5-2450, 2.50GHz and 23.4 GB RAM, our more complex model that follows the approach described in Sections 3.2, 3.3, and 3.4 took approximately 52 min.

The BUGS models, the R codes as well as the simulated data sets presented in the following section (along with the shapefiles) are available in the Supporting Information and in Figshare (https://figshare.com/projects/Simulated_data_and_codes_for_the_generalized_EPS/64382). The lung cancer cases and HSfE data used in the application are not available for sharing because they contain potential personally identifiable information.

## 4 Simulations

To emulate a real-world spatial domain *𝒟* = {1,…, *N*}, but at the same time to maintain reasonable computing time, we construct the simulation study over London decomposed into 625 electoral wards. The generation of the data is structured in three consecutive steps, namely, (i) configuration of spatial dependencies described using exponential covariance functions, (ii) simulation of the ecological-scale variables, and (iii) simulation of the individual-scale variables.

We consider three simulation designs, defined by three assumptions about the spatial coverage of the individual-level variables and the associated missingness mechanism: (i) *simulation design 1* with a full spatial coverage, (ii) *simulation design 2* with sparse spatial coverage under MAR mechanism, and (iii) *simulation design 3* with sparse spatial coverage under MNAR mechanism. To get the incomplete spatial coverage in ***𝒟***, two binary missing data indicators are constructed, which are successively applied to the complete data design. For both designs, we assume the individual-level data are missing in approximately 50% of London’s wards. For each simulation design, we explore two scenarios where the generalized EPS and the health outcome are linearly and nonlinearly related on the log scale. All results are averaged across 100 simulated data sets for each scenario.

### 4.2 Design

First, we define neighbors as areas within a certain distance of each other and we generate two independent spatial processes, ***ζ**_i_* = (*ζ*_*i*1_, *ζ*_*i*2_)^⊤^, using a geostatistical model. In detail, we compute a symmetric matrix *D_is_* with entries *d_is_* corresponding to the distances between ward centroids *i* and *s*, and we simulate two independent Gaussian processes with zero mean and covariance function defined by an exponential model **σ**^2^exp(−***ψ***‖*d*‖). Here, $\sigma^{2}=(\sigma_{1}^{2},\sigma_{2}^{2}{)}^{⊤}$ are constant variance parameters, ‖*d*‖ is the distance (norm) between the centroids of the wards, and **ψ** = (*ψ*_1_, *ψ*_2_)^⊤^ is the parameter that controls the rate of decay of the correlation as the distance between sites increases (Banerjee, Carlin, & Gelfand, 2014). Using different parameterizations for *ψ*_1_ and *ψ*_2_, we ensure a substantial amount of spatial correlation, allowing for an effective range (i.e., the distance at which the covariance becomes negligible, dropping to 0.05, computed as −log(0.05)/**ψ**) between 15 and 25 km (note that the range of distances lies approximatively between 0.5 and 53 km). Subsequently, we simulate the following synthetic ecological data:

(i) A continuous confounder, *C_i_*, that we assume to be known and measured over the geographical domain. We simulate *C_i_* from an independent Gaussian distribution, with mean centered on *ζ*_*i*1_ and relatively small variance (i.e., 0.5) to ensure we retain the spatial structure.
(ii) *K*(=5)-dimension correlated mixed-type confounding variables, **M**_*i*_ = (*M*_*i*1_, …,*M*_*i*5_), that we assume to be unmeasured. We simulate these variables using a joint model generated by a copula, which is in extreme synthesis a joint cumulative distribution function (cdf) of random variables with uniformly distributed margins on the unit interval (e.g., Nelsen, 2006). The advantage of using a copula is that it allows to capture the pure dependence between random variables without the influence of the marginal distributions. Here, to couple marginal distributions, we adopt a Gaussian copula using the copula package in R. We simulate *M*_*i*1_, …*M*_*i*5_ specifying a correlation structure between these variables as follows:
  $${\text{R}}_{\text{M}}=\left(\begin{array}{ccccc} 1 & \; & \; & \; & \;\\ 0.7 & 1 & \; & \; & \;\\ 0.3 & 0.2 & 1 & \; & \;\\ 0.2 & 0.1 & 0.4 & 1 & \;\\ 0.2 & 0.1 & 0.4 & 0.5 & 1 \end{array}\right)$$
  They belong to the following distributions: Gaussian(*M*_*i*1_,…, *M*_*i*3_) and Beta(*M*_*i*4_ and *M*_*i*5_). In detail, we fix the parameters of these marginal distributions as follows: *M*_*i*1_ ~ N(0,1), *M*_*i*2_ ~ N(0,1), *M*_*i*3_ ~ N(*ζ*_*i*2_,1), *M*_*i*4_ ~ Beta(12,12), *M*_*i*5_ ~ Beta(6,14). Taking Beta densities for *M*_*i*4_ and *M*_*i*5_ with the specified shape parameters, we ensure a mean of 0.5 and 0.3, respectively.
(iii) A continuous variable defining the exposure of interest, *X_i_*. We simulate the exposure variable from a Gaussian distribution:
  (6) $$X_{i}∼\text{N}(0+C_{i}0.3+M_{i1}0.2+M_{i2}0.3+M_{i3}0.4+M_{i4}0.1+M_{i5}0.1,0.25).$$
  Note that to induce a spatial structure in *X_i_*, we use stronger coefficients for the two variables that are spatially structured, that is, *C_i_* and *M*_*i*3_.
(iv) A count variable of health outcomes, *Y_i_*. We generate the health data from a Poisson distribution:
  (7) $$Y_{i}∼\text{Poisson}(E_{i}\;\text{exp}(0+X_{i}0.2+C_{i}0.2+M_{i1}0.2+M_{i2}0.2+M_{i3}0.2+M_{i4}0.2+M_{i5}0.2+\theta_{i}),$$
  where *θ_i_* ~ N(0,0.05) and the expected number of cases in each ward is generated from a uniform distribution in [15–30].

Then, we turn to the generation of the individual-level data. We assume to have 20 subjects with available information in each area *i*, for *j* = 1, …, *J_i_*. We generate five variables for each subject *j* in each area *i*, **m**_*ij*_= (*m*_*ij*1_,…, *m_ijK_*), for *k* = 1, …, *K* = 5, from a copula structure holding the same correlation structure used to generate the **M**_*i*_. The marginals for the three Gaussian individual-level variables, *m*_*ij*1_,… *m*_*ij*3_, are centered on the mean of the three Gaussian ecological variables *M*_*i*1_,…, *M*_*i*3_. The marginals of the remaining two individual-level variables, *m*_*ij*4_ and *m*_*ij*5_ are discrete Bernoulli distributions with probability of success set to be equal to the mean of the Beta distributed *M*_*i*4_ and *M*_*i*5_.

### 4.3 Sparsity of individual-level data

We generate two binary missing value indicators, *l* ∈ {MAR, MNAR} for *i* = 1, …, *N* such that:

$$\ell_{i}^{l}=\{\begin{array}{l} 0\;\text{if}\;m_{ijk}\;\text{is}\;\text{observed}\;\\ 1\;\text{if}\;m_{ijk}\;\text{is}\;\text{missing}\; \end{array}$$

from Bernoulli distributions with probabilities defined as follows:

- MAR design: $\text{P}(\ell_{i}^{\text{MAR}\;}=1|X_{i},C_{i})=\frac{\text{exp}(-0.4+X_{i}0.6+C_{i}0.5)}{1+\text{exp}(-0.4+X_{i}0.6+C_{i}0.5)},$
- MNAR design: $\text{P}(\ell_{i}^{\text{MNAR}\;}=1|X_{i},C_{i},Z_{i}^{\text{True}\;})=\frac{\text{exp}(-0.9+X_{i}0.7+C_{i}0.7+Z_{i}^{\text{True}\;}1)}{1+\text{exp}(-0.9+X_{i}0.7+C_{i}0.7+Z_{i}^{\text{True}\;}1)}.$

Here, $Z_{i}^{\text{True}\;}$ is the linear combination of **M**_*i*_, with coefficients specified in (6). Then, the two simulated indicators are used to distinguish the survey areas *S* from those with no individual-level data, forming the MAR and MNAR simulation designs.

### 4.3 Nonlinearity

We explore the effect of a nonlinear relationship between the health response *Y_i_* and the unmeasured confounders **M**_*i*_ summarized in the generalized EPS within the three simulation designs, fitting a polynomial model with quadratic terms for *M*_*i*,1:2_ for generating the outcome data in (7). More specifically, the quadratic polynomial has the form: $M_{i1}0.2+M_{i1}^{2}0.2$ and $M_{i2}0.2+M_{i2}^{2}0.2$.

### 4.4 Model comparison and performance metrics

Within the three simulation designs, we compare the performance of our approach under linear and nonlinear scenarios. Regarding the nonlinear scenario, we parameterize *f*() as a quadratic polynomial. Then, we explore the effect of forcing *f*() to be a linear function, although the relationship between **Y** and several **M**_*i*_ is expressed via a quadratic polynomial.

Within the simulation design 1, which assumes a full geographical coverage of the individual-level data, we compare also the results of our approach with those obtained from: (i) a “true” (benchmark) model, that assumes the availability of all confounders **M**_*i*_, allowing us to evaluate the potential information loss due to inclusion of the generalized EPS *Z_i_* instead of the actual confounders, and (ii) a naïve ecological model, that does not consider the individual-level data, allowing us to evaluate the bias due to missing confounders.

We fixed the regression coefficient associated with the exposure, that is, *β_X_* = 0.2, as specified in (7), and we term this as the target value. Then, we investigate the ability of our approach to recover the target value in terms of bias, precision, and accuracy. In particular, by comparing, at each MCMC iteration *r*, the posterior estimate of *β_X_* with the target value, we compute the following performance metrics: (i) the mean bias (MB), that is the mean of the differences between the estimated coefficient at each iteration *r* and the target value; (ii) the root mean squared error (RMSE), that is the square root of the mean of the squared differences between the estimated coefficient at each iteration *r* and the target value; (iii) the coverage of the nominal 95% posterior credible intervals (CIs), that measures the percentage of times that the target value is included within the CI of the estimated coefficient, and (iv) the width of the 95% CI.

### 4.5 Simulation results

We carried out graphical checks for the simulation designs 2 and 3 to inspect the relationship between the “true” generalized EPS obtained as the linear combination of **M**_*i*_ and the coefficients specified in (6), and the imputed generalized EPS for the out-of-sample areas in the analysis stage. We found that our imputation model is able to predict the generalized EPS accurately. In the Supporting Information (Section S2.2), we report the plots for simulation designs 2 and 3 using a randomly selected data set.

Table 1 presents the posterior mean of estimated regression coefficient *β_X_* and the performance metrics for each scenario within the three simulation designs.

Simulation design 1 assumes a full spatial coverage of the individual-level data, and results are obtained for the true benchmark model (assuming that the ecological-level variables **M**_*i*_ are actually known), the naïve ecological model and the health outcome model that includes the generalized EPS. Under a linear scenario, which assumes a linear relationship between the up-scaled variables **M**_*i*_ and the outcome **Y**, simulation design 1 shows that ignoring the individual-level data (i.e., naïve analysis model), the health-effect estimates exhibit large bias, with a drop of the coverage and a high RMSE. Moreover, it confirms that the health-effect estimated via generalized EPS adjustment is competitive with the one obtained via direct inclusion of the **M**_*i*_ as confounders (i.e., true benchmark model), showing a minimum of information loss using the generalized EPS. This is in line with the finding by Wang et al. (2019) related to the adjustment via EPS when the exposure variable is binary. Similar results are observed on the nonlinear scenario. It is also noticeable that assuming the log relative risk log(*μ_i_*) to be linear in all its predictors does not seem to lead to bias due to a misspecification of the relationship between *Z_i_* an *Y_i_*, however the width of the 95% CI is larger in comparison to the more flexible specification of a quadratic polynomial for *Z_i_*.

Table 1 also demonstrates the performance of the approach under the remaining simulation designs, where the individual-level data **m**_*ij*_-are not fully observed (i.e., *i* ∈ *S*) under assumptions of MAR and MNAR mechanisms.

In simulation design 2, a complete case analysis under linear scenario between the *Y_i_* and the **M**_*i*_ tends to increase the bias in the health-effect estimates slightly. In the presence of nonlinearity between the *Y_i_* and the **M**_*i*_, assuming a linear relationship does not seem detrimental to the estimates, suggesting that the nonlinearity does not necessarily transfer to the relationship between generalized EPS and health outcome.

In simulation design 3, the generalized EPS adjustment seems to lead to a better performance in comparison to a complete case analysis. In particular, under a nonlinear scenario between the *Y_i_* and the **M**_*i*_, we notice that a linear approximation in (4a) for *f*() increases the MB in the posterior estimates and leads also to a larger RMSE and lower coverage. Here, the flexibility in the functional form for *f*() seems to help, and we assume this is due to the increased complexity due to the additional missingness model. These results support the necessity to investigate the relationship between the health outcome and the generalized EPS (in the in-sample areas) before to specify the functional form for *f*().

In the Supporting Information (Section S2.3), we present results from additional simulation studies with 30% of sparsity for the individual-level potential confounders, fixing *β_X_* to either 0.20 or 0.00. Moreover, we present the results from a simulative example where **X** and **Y** variables are generated using different and more diverging parameters (Section S2.4).

## 5 Lung Cancer Mortality and NO_2_ Exposure Analysis

We present now the modeling details and the results obtained by applying the newly proposed approach to the case study introduced in Section 2. The notation used to describe the methodological approach is aligned with the data available for the case study as follows:

- The area-referenced observed health outcome **Y** and exposure **X** are given by the lung cancer mortality counts and the ambient NO_2_ concentrations, respectively.
- The area-referenced observed covariates **C**_*i*_ are given by the Carstairs index and the average of home radon measurements.
- The individual-level observed potential confounding variables available by a sample survey **m**_*ij*_, where *i* = 1, …, *S* ∈ *N*, are given by HSfE data.

The latent quantities **M**_*i*_ (i.e., the unobserved latent potential confounders at area-level) are obtained through the upscaling on the areas covered by HSfE and consequently the generalized EPS is computed. Because HSfE provides a sparse spatial coverage, so that the generalized EPS covers only the 80.7% of the England’s LADs, we adopt an imputation step to predict it for the out-of-sample LADs. In comparison with the naïve ecological model described in (1), this allows a fuller adjustment for confounding of the risk estimates.

### 5.1 Modeling details

The design stage, related to the up-scaling of HSfE data and the construction of the generalized EPS, is developed as described in Sections 3.2 and 3.3. After completion, we observed a nonlinear relationship between the generalized EPS (computed for the LADs covered by the HSfE) and the home radon measurements, as well as between the generalized EPS and the lung cancer SMRs (see Supporting Information, Section S1.4). We accommodated nonlinearity by using spline functions. Additionally, to improve convergence in the analysis stage of our approach, we centered NO_2_ concentrations.

With reference to the imputation model for the generalized EPS, as discussed by White and Carlin (2010), it is not possible to distinguish which missingness assumption is appropriate from the observed data. However, using some data visualization and contextual knowledge we consider that a MAR mechanism is plausible for our case study. In particular, the pattern displayed by the ecological-level data (i.e., deprivation index and home radon measurements) was found reasonably similar between LADs where the individual-level data from HSfE were available and the others where these data were missing (see Supporting Information, Section S1.5). In addition, because HSfE is conducted via a random selection of the address of people living in private households in England (see Section 2.2), meaning every address had an equal chance of being included, we excluded a MNAR mechanism, being unlikely that the probability of missingness of the generalized EPS (which is estimated upon the individual-level data) depended on the values of the individual-level variables missing in the HSfE samples.

### 5.2 Results

We compare the results from our proposed modeling approach with those obtained from: (i) the naïve Poisson log-linear model as described in (1), and (ii) the complete case analysis approach, which considers only the England’s LADs where HSfE data are available.

Without considering the individual-level risk factors, the naïve ecological model suggests a negative association between lung cancer mortality and NO_2_ with considerable uncertainty. In particular, on average, there is a decrease of −1.46% (95% CI: −5.14% to 2.34%) in lung cancer mortality associated with a 10-μg m^−3^ increase in NO_2_ exposure, with only a small posterior probability (equal to 0.23) that the mortality risk is greater than one. The counterintuitive result suggests a possible bias due to residual confounding.

Considering only the LADs covered by the survey (i.e., complete case analysis) and augmenting the health-effect model with the latent construct provided by the individual-scale variables via generalized EPS, the direction of the association is reversed. The change in lung cancer mortality per 10-μg m^−3^ increase in NO_2_ is 6.98% (95% CI: 2.42% to 11.89%). The posterior probability that the mortality risk is greater than one is 0.99, pointing to conclusive evidences of a cancer risk associated with NO_2_.

The approach applied on the whole of England (i.e., including generalized EPS imputation and adjustment) estimates an increase in lung cancer mortality of 4.82% (95% CI: −0.28% to 10.54%). The posterior probability that the mortality risk is greater than one becomes 0.97. Comparing the performance of our proposed approach with the complete case analysis, we observe more uncertainty in the effect estimated by the former, as it reflects the uncertainty in the EPS imputation. In particular, as in the analysis stage the imputation and outcome models are run jointly, uncertainty about the EPS imputation is automatically accounted for in the outcome model.

**Figure 3:**
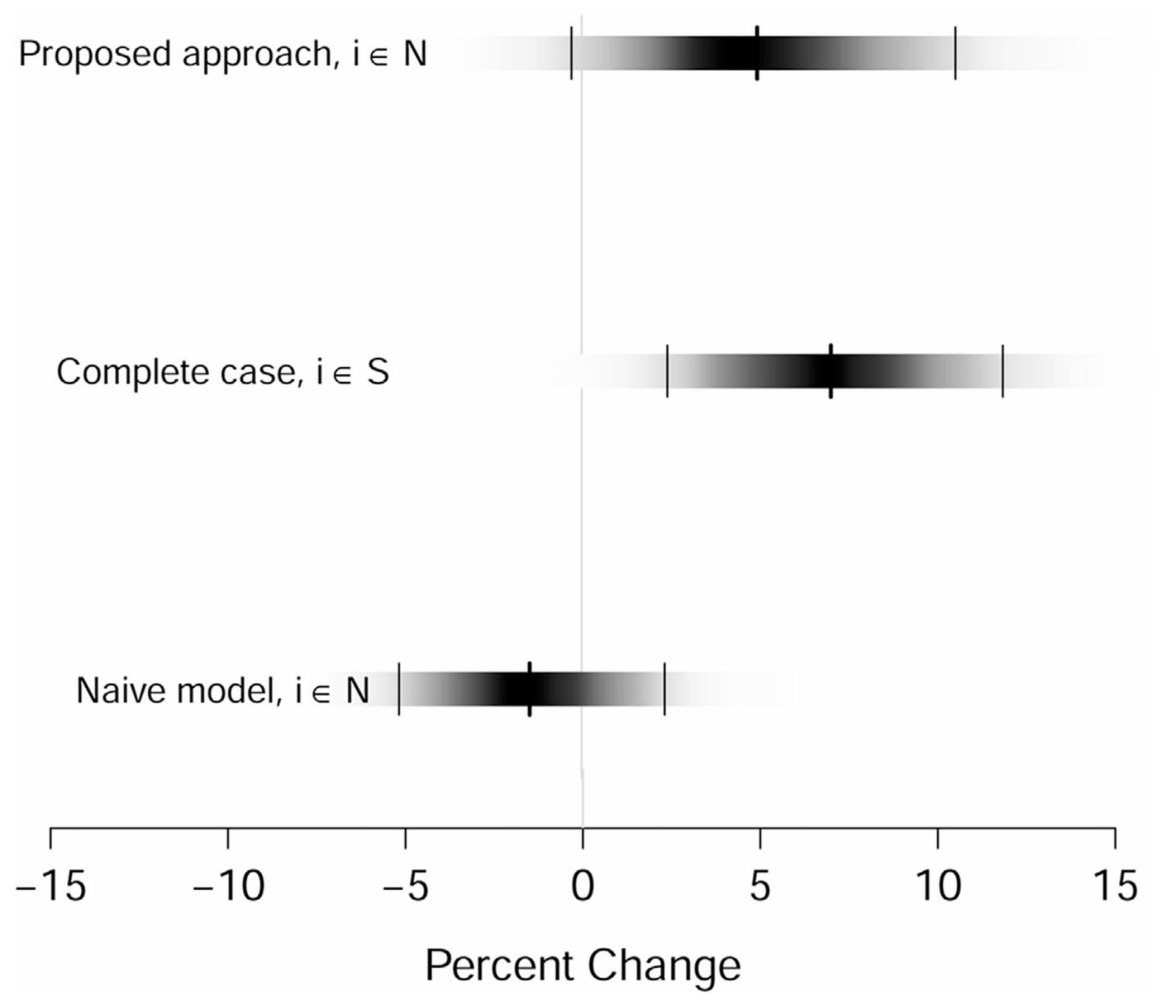
Plot of the full posterior distribution (shaded rectangular strips) of the percent change in the relative risk of lung cancer mortality associated with an increase of 10-μg m^−3^ in NO_2_ concentrations for the naïve model, the complete case analysis and the analysis performed according to our proposed approach. The darkness of the strips is proportional to the density, such that the strip is darkest at the maximum density and fades to white at the minimum density

Figure 3 depicts the posterior distribution of the percent change estimates from our analyses, where the darkness of the strips at a point is proportional to the posterior density of the estimated parameters (Jackson, 2008). A recent meta-analysis of observational cohort and case-control studies by Hamra et al. (2015) quantified a change in lung cancer mortality associated with a 10-μg m^−3^ increase in NO_2_ of 4% (95% CI: 1%, 8%). The result obtained from our proposed approach in Figure 3, is consistent with Hamra et al. (2015)’s meta-estimate, albeit being characterized by larger uncertainty.

Table 2 shows the posterior means for the fixed regression parameters from the analysis performed following our proposed approach, estimating the effects of the ecological-level covariates on lung cancer mortality risk.

As expected, we found evidence that increasing levels of deprivation (measured by the Carstairs index) are associated with an increased lung cancer mortality risk. Comparing the higher quintiles with the lowest quintile of the index of deprivation (which defines the least deprived category), we found that the 95% CIs of the estimated parameters do not include the null risk of zero. In particular, the areas most deprived have 1.73 (i.e., exp(0.55)) times the mortality rate of the least deprived areas. No excess of lung cancer mortality was observed in relationship to the average measurements of radon.

## 6 Discussion

In this paper, we have described a comprehensive methodological framework for residual confounding adjustment in area-referenced studies via generalized EPS, with the aim of improving inference in estimating the health risks associated with potential environmental threats. We showed that by capturing the ecological-scale behavior of unmeasured confounders by locally sampling individual-scale data summarized in a continuous composite score, the bias due to residual confounding is alleviated. Area-referenced studies have been often criticized for being associated with uncertainty and information loss due to data aggregation (e.g., Greenland, 2001; Greenland & Morgenstern, 1989). Therefore, we believe that our proposed methodology provides a promising tool for reducing the bias due to unmeasured confounding in ecological environmental studies.

Using a realistic case study, we showed the applicability of our approach in empirical research. In particular, we focused on determining whether exposure to NO_2_, which is a gaseous pollutant used as a marker of traffic pollution, is associated with lung cancer mortality in England. Primary NO_2_ forms when fossil fuels (such as coal, oil, gas, or diesel) are burned at high temperatures, while secondary NO_2_ forms in the atmosphere by the oxidation of nitrogen oxide. Until recently, the range of negative health effects associated with NO_2_ has been thought to be likely imputable to other toxic pollutants related with it, such as its reaction products including ozone and secondary particles (e.g., WHO/Europe, 2013). Even so, recent literature has suggested that NO_2_ could play a direct causal harmful role for itself (e.g., Faustini, Rapp, & Forastiere, 2014; Walton et al., 2015). Our study contributes insights into the complex relationship between long-term exposure to NO_2_ and lung cancer risk. We found a positive association between cancer mortality risk and NO_2_ exposure (considering a latency period of 10 and 11 years) when the ecological area-referenced models are adjusted for the individual-level survey’s data through the generalized EPS. This was consistent in both the complete case analysis (i.e., where the approach is restricted only to the areas covered by the individual-level data) and in the full analysis (i.e., where the approach is extended to the entire geographical domain under study), although the latter presented an increase in the uncertainty due to the imputation model for the generalized EPS in the areas where the individual-level data were not available. Importantly, our ecological parameter’s estimate of the effect of NO_2_ exposure is in line with the individuallevel estimation provided by a meta-analysis on cohort and case-control studies by Hamra et al. (2015), as pointed out in Section 5.

In our application, we adjusted the health-effect estimate using also freely available home radon concentrations described by their arithmetic average per LAD upon a 35-year period. This is a crude indicator, and the use of more precise measurements could increase the performance of our approach. A further limitation is given by the fact that we did not have information on residential mobility patterns of cancer’s cases, which may have contributed to exposure misclassification. In this regard, we would like however to point out that in a long-term study performed on 367,658 individuals, followed from 1971 to 2009, to assess the effect of air pollution (estimated at residence) on mortality in England and Wales, Hansell et al. (2016) found that restricting the analysis to the nonmovers (in the five years prior to the 1971 census) made no difference in risk estimates.

In our simulation study and real application, we accommodated the spatial nature of the data using random effects with priors modeled using CAR processes. We based their formulation on distance-based weight matrices (Duncan, White, & Mengersen, 2017). Because individual-level spatially referenced data from surveys were sparse and missing in several areas, we considered that a distance-based neighborhood structure represents a more convenient solution in comparison to a simple adjacency structure. In this study, we assumed the same weights for the nearest neighbors of each area. A more flexible solution could be obtained using a tapering function based on the nearest neighborhood structure (Lee, 2018), even if it would increase the computational burden of our approach. Moreover, this would work if there is evidence that areas close to each other are not all similar. An alternative solution to account for the spatial sparsity in the sampled individual-level variables may be given by the approach used by Wang et al. (2019), which considered a super-layer geographical setting for the up-scaling and imputation model. We explored both the approaches (i.e., distance-based structure vs. adjacency on super-layer spatial structure) within our simulation studies, and the distance-based neighborhood weight matrix resulted in a better estimation of the ecological unmeasured confounders, that is, **M**_*i*_. This consequently led to a better estimate of the generalized EPS and a lower bias in estimating the parameter quantifying the health-effect associated to the exposure of interest *β_X_* (results not shown).

We explored how the generalized EPS can be used under MAR and MNAR missingness mechanisms; for the latter, we exploited sensitivity analyses to check the robustness of the estimate of the regression parameter *a_z_* within the missingness model, due to the lack of information from the data. From the results obtained in these simulated analyses (see Supporting Information, Section S2.1), it is apparent that the imputation methodology under MNAR assumption is reasonably robust. Unless prior knowledge about the missingness mechanism exists, we strongly recommend the readers to perform sensitivity analyses for this parameter, exploring a wide range of potential values, which then are used to specify an informative prior.

Methodologically, our key modeling strategy relies on a sequential two-stage Bayesian framework, where parameter estimates in the outcome model are made conditional on observed potential confounders and on the generalized EPS obtained from a precedent design stage, where it is anchored upon in-sample survey areas. Our strategy implies a propagation of uncertainty from the design stage to the analysis stage, but no “feedback” between these two stages (i.e., the estimation of generalized EPS in the design stage is separated from the health-effect model in the analysis stage). This was reached using a probability model for the generalized EPS in the analysis stage, where we account for the uncertainty associated with its estimation in the design stage. Recently, Zigler (2016) discussed the incorporation of the propensity score into an outcome model from a Bayesian perspective and underlined a still existing discordance in literature between the need for a fully Bayesian analysis and the necessity of cutting the feedback of information between the propensity score estimation and the outcome analysis model to produce a reliable estimate of health effects. In our work, we choose a simple yet effective two-stage separable Bayesian approach with uncertainty propagation via probabilistic model for the estimated generalized EPS, but we acknowledge that this would be an important area for further research.

A key aspect linked to the analysis stage is given by the empirical evaluation of the functional form of the generalized EPS–health response relationship. Our study points out that care should be taken in this regard, as a misspecification of such relationship could lead to biased estimates. This supports previous analyses performed on individual-level–based studies (e.g., Kreif, Grieve, Díaz, & Harrison, 2015). Therefore, we would like to stress the need to identify flexible and data-adaptive ways for capturing such relationship.

Overall, in this paper, we have provided (i) a generalization of the propensity score methodology to ecological area-referenced studies when the exposure of interest is continuous, (ii) a method to deal with unknown confounders that are potentially nonlinear in their relationship with the health outcome, (iii) a simple way to propagate uncertainty from the design stage to the analysis stage, and (iv) a procedure for specifying the imputation model for the generalized EPS under different assumption of missingness mechanisms. The benefit of our approach is that it is practical and reproducible and, although motivated by specific applications in environmental epidemiology, the generalized EPS strategy we have outlined here is broadly applicable in other contexts.

In this paper, we rely on parametric model assumptions in estimating the generalized EPS, but other techniques could potentially accomplish similar results (Westreich, Lessler, & Funk, 2010). Therefore, further directions of our work include the evaluation of nonparametric approaches for the generalized EPS model. Moreover, in this work, we did not use sampling survey weights in the up-scaling of the individual-level potential confounders, which however may be considered to alleviate bias due to nonresponse and nonrandom sampling (Mercer, Wakefield, Chen, & Lumley, 2014). The incorporation of such sampling weights is out the scope of the current paper, but this would be a point deserving future investigation. Finally, while in this paper we framed the inference at ecological scale, being bounded by confidentiality reasons that in this instance did not allow us to collect spatial residential coordinates of the cancer cases, a new study is in course of ratification, where geolocated health outcome data are measured at individual level, while measurement of environmental exposure will be available at aggregated level. This will potentially allow for a semiecological design that will take advantage of downscaling models for the exposure to capture localized behavior of environmental factors and a log-Gaussian Cox process within the analysis stage of our methodological approach.

## Supplementary Material

Supplementary File 1

Supplementary File 2

Supplementary File 3

## Figures and Tables

**Table 1:** Parameter estimation for *β_x_* in the three simulation designs, where **m**_*ij*_ are assumed fully observed (design 1) and spatially sparse respectively under a MAR (design 2) and MNAR (design 3) mechanisms (target value *β_x_* = 0.20). Posterior mean (95% credible interval (CI)), mean bias (MB), root mean square error (RMSE), coverage and width of 95% CI. Note that *N* refers to the total number of areas, while *S* refers to the subset of the areas with survey data. Results from 100 replicated data sets

Models	Post. mean (95% CI)	MB	RMSE	Coverage	Width
**Simulation design 1:**					
**All the variables are fully observed**					
*Linearity between* *Y_i_* *and* **M**_*i*_					
True (benchmark) model	0.20 (0.13 to 0.27)	0.00	0.04	95%	0.11
Naïve model	0.80 (0.76 to 0.83)	0.60	0.60	0%	0.05
*Z_i_* adj	0.21 (0.12 to 0.29)	0.01	0.04	94%	0.12
*Nonlinearity between* *Y_i_* *and several* **M**_i_					
True (benchmark) model	0.20 (0.14 to 0.27)	0.00	0.04	95%	0.09
Naïve model	0.82 (0.70 to 0.94)	0.62	0.62	0%	0.11
*Z_i_* adj via polynomial function	0.19 (−0.02 to 0.39)	−0.01	0.11	87%	0.26
*Z_i_* adj via linear function	0.20 (−0.05 to 0.45)	0.00	0.13	90%	0.31
**Simulation design 2:**					
**Sparsity under MAR mechanism**					
*Linearity between* *Y_i_* *and* **M**_*i*_					
*Z_i_* adj, *i* ∈ *S* (complete case analysis)	0.24 (0.09 to 0.38)	0.04	0.09	72%	0.16
*Z_i_* imp & adj, *i* ∈ *N*	0.20 (0.08 to 0.34)	0.00	0.06	91%	0.16
*Nonlinearity between* *Y_i_*, *and several* **M**_*i*_					
*Z_i_* adj, *i* ∈ *S* (complete case analysis)	0.24 (−0.05 to 0.51)	0.04	0.15	96%	0.41
*Z_i_* imp & adj via polynomial function	0.25 (−0.03 to 0.51)	0.05	0.15	70%	0.28
*Z_i_* imp & adj via linear function	0.19 (−0.17 to 0.57)	−0.01	0.19	81%	0.43
**Simulation design 3:**					
**Sparsity under MNAR mechanism**					
*Linearity between Y. and* **M**_*i*_					
*Z_i_* adj, *i* ∈ *S* (complete case analysis)	0.22 (0.07 to 0.39)	0.02	0.08	86%	0.19
*Z_i_* imp & adj, *i* ∈ *N*	0.20 (0.06 to 0.37)	0.00	0.07	94%	0.18
*Nonlinearity between* *Y_i_* *and several* **M**_*i*_					
*Z_i_* adj, *i* ∈ *S* (complete case analysis)	0.28 (−0.03 to 0.59)	0.08	0.18	78%	0.38
*Z_i_* imp & adj via polynomial function	0.22 (−0.03 to 0.43)	0.02	0.12	83%	0.27
*Z_i_* imp & adj via linear function	0.36 (0.02 to 0.85)	0.16	0.26	56%	0.40

**Table 2:** Estimation of the effects of the ecological-level covariates on lung cancer mortality. Posterior mean and 95% credible interval (CI)

Ecological-level covariates	Post. mean	95% CI
Carstairs index (in quintiles)		
1 (least deprived)	Ref	
2	0.14	0.07 to 0.20
3	0.21	0.15 to 0.27
4	0.36	0.30 to 0.42
5 (most deprived)	0.55	0.48 to 0.62
Radon in homes (average)	0.00	−0.02 to 0.01
