# Supplementary material for: A flexible hierarchical framework for improving inference in area-referenced environmental health studies [image]
Source: Biom J. Author manuscript; Available in PMC 2022 Dec 20. (PMC7613972; doi:10.1002/bimj.201900241)
Supplement: Supplementary File 1 [file EMS158440-supplement-Supplementary_File_1.docx]

**Supporting Information**

Please upload the Codes & Data folder from:

<https://www.dropbox.com/s/on25jfgetxw4j45/CodeData.zip?dl=0>
