# Supplementary material for: A flexible hierarchical framework for improving inference in area-referenced environmental health studies [image]
Source: Biom J. Author manuscript; Available in PMC 2022 Dec 20. (PMC7613972; doi:10.1002/bimj.201900241)
Supplement: Supplementary File 2 [file EMS158440-supplement-Supplementary_File_2.pdf]

SUPPORTING INFORMATION
A flexible hierarchical framework for improving inference in area-referenced environmental health studies

Monica Pirani[1], Alexina J. Mason[2], Anna L. Hansell[3], Sylvia Richardson[4], and Marta Blangiardo[1]

[1] MRC Centre for Environment and Health, Department of Epidemiology and Biostatistics, Imperial College London, London W2 1PG, UK
[2] Department of Health Services Research and Policy, London School of Hygiene and Tropical Medicine, London WC1H 9SH, UK
[3] Centre for Environmental Health and Sustainability, University of Leicester, Leicester LE1 7HA, UK
[4] MRC Biostatistics Unit, Cambridge Institute of Public Health, University of Cambridge, Cambridge CB2 0SR, UK

Contents

S1 Additional material for the real case study **2**
- S1.1 Details of the land use regression (LUR) model used to obtain the concentrations of NO_2 for England . . . 2
- S1.2 Scatterplot of lung cancer standardized mortality ratios (SMRs) *vs* NO_2 . . . 2
- S1.3 Map of the two ecological-level covariates . . . 3
- S1.4 Relationship between the estimated generalized EPS with (i) the home radon measurements, and (ii) the SMRs . . . 3
- S1.5 Distribution of the ecological-level covariates within LADs covered and uncovered by HSfE . . . 4

S2 Additional material for the simulation study **5**
- S2.1 Sensitivity analysis to evaluate identifiability of the imputation model under the assumption of missing not at random (MNAR) mechanism for the generalized EPS . . . 5
- S2.2 Checking plots for the generalized EPS . . . 6
- S2.3 Additional simulations assuming a 30% of sparsity for the individual-level potential confounders and assuming different health effect sizes . . . 6
- S2.4 Additional simulations using diverging parameters for generating $\mathbf{X}$ and $\mathbf{Y}$ variables . 9

S1 Additional material for the real case study

S1.1 Details of the land use regression (LUR) model used to obtain the concentrations of NO_2 for England

The case study relies on a LUR model for the NO_2 (μg m^{-3}) exposure data for the year 2001. The details regarding this multivariate regression model, built on Geographic Information System (GIS)-based covariates, are described in Vienneau *et al.* (2010). Briefly, the model was constructed on the annual mean concentrations of NO_2 obtained from the national air quality network and supplemented with data from networks run by local authorities, involving a total of 156 monitoring sites across Great Britain. Predictor variables used for modeling were related to land cover, population, traffic intensity, road network and topography characteristics within buffers. The data on these predictor variables were integrated in a GIS, and converted to raster, which is a spatial data structure dividing Great Britain into square grids of 100m $\times$ 100m resolution, called cells. The map of the gridded NO_2 data is displayed in Figure S1.

Figure S1: Map of NO_2 concentrations from LUR for Great Britain, year 2001.

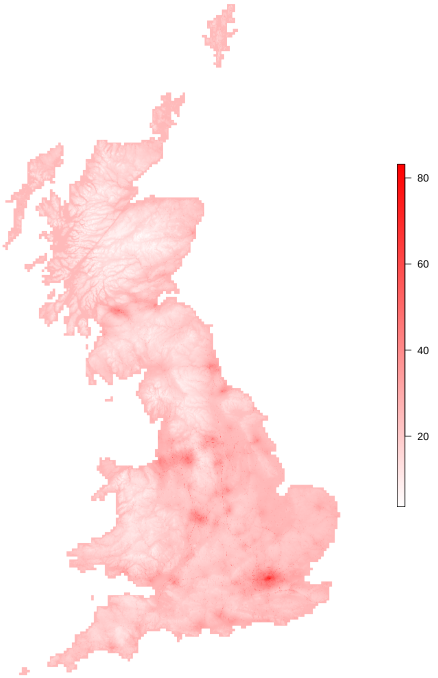

Thus, because NO_2 values from LUR were in regular spatial grid, we converted the raster structure of the data to a vector feature output. To do so, we overlaid the ESRI shapefile with polygons (areas) corresponding to the England's local authority districts (LADs) to the Great Britain grid map. A NO_2 value of a grid cell was considered contributing to a polygon if falling (completely or partially) within the polygon boundary. A weighted mean function was finally specified over the values falling in each polygon, where the weighs were represented by the proportion of the grid included in the polygon boundary.

We used the `R` packages `raster` and `rgdal` to process the NO_2 exposure data.

S1.2 Scatterplot of lung cancer standardized mortality ratios (SMRs) *vs* NO_2

Figure S2 displays the relationship between the SMRs on natural logarithm and NO_2 concentrations on square root scale, which seems to be linear.

Figure S2: Scatterplot of lung cancer SMRs (on log scale) *vs* NO_2 concentrations (on square root scale).

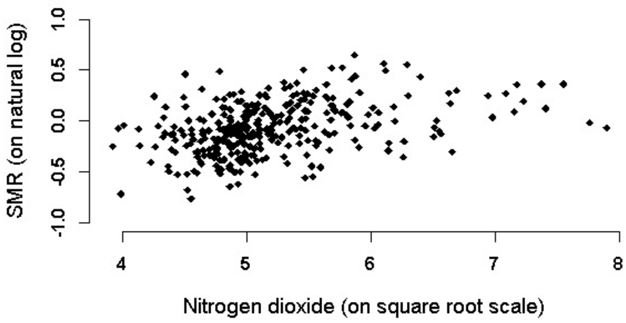

S1.3 Map of the two ecological-level covariates

Figure S3 presents the maps of the two LAD-level covariates used in the real case study. They are (i) the Carstairs index, which is used as measure of deprivation and displayed from least deprived LADs in England (quintile 1) to most deprived (quintile 5); and (ii) the indoor radon measurements (Bq m^{-3}), which are synthesized by the arithmetic average of the home radon levels in each LAD.

Figure S3: Maps of the Carstairs index (Panel A), and the average radon concentrations (Panel B).

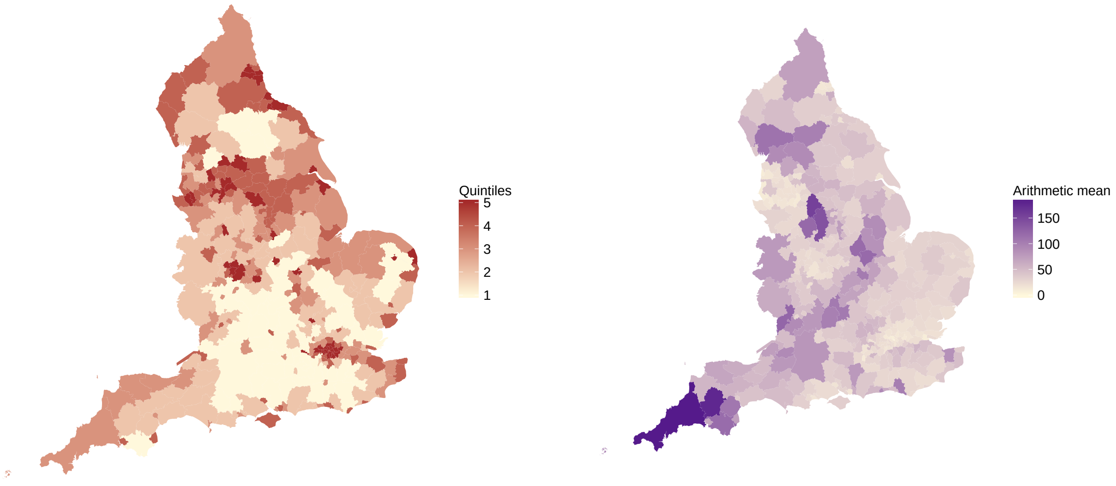

Panel A

Panel B

S1.4 Relationship between the estimated generalized EPS with (i) the home radon measurements, and (ii) the SMRs

Figure S4 presents the (nonlinear) relationship between the posterior mean of the generalized EPS constructed on the LADs covered by the Health Survey for England (HSfE) and other variables within the analysis stage of the real case study. In particular, the plots display (i) the relationship between the generalized EPS and the square-root of the home radon measurements, and (ii) the relationship between the generalized EPS and the SMRs on natural log scale.

Figure S4: Relationship between the generalized EPS (posterior mean) with the square-root of the home radon measurements (Panel A), and the generalized EPS (posterior mean) with the SMRs (Panel B).

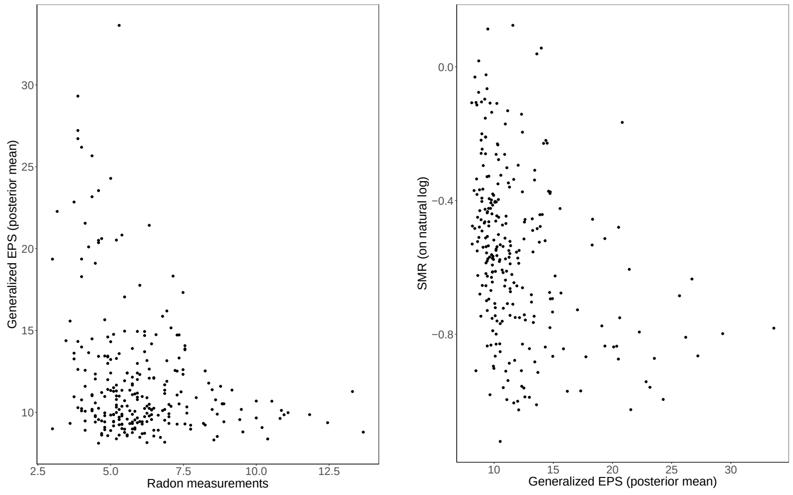

Panel A Panel B

S1.5 Distribution of the ecological-level covariates within LADs covered and uncovered by HSfE

Figure S5 presents the distribution of the two ecological-level covariates, i.e. Carstairs index and home radon measurements comparing those in the LADs where HSfE data are observed (in-sample areas) and those where HSfE data are missing (out-of-sample areas). We can appreciate minimal differences in the distribution of the two ecological-level covariates between in-sample and out-of-sample areas.

Figure S5: Distribution of Carstairs index (Panel A) and home radon measurements (Panel B) between LADs covered and uncovered from HSfE data.

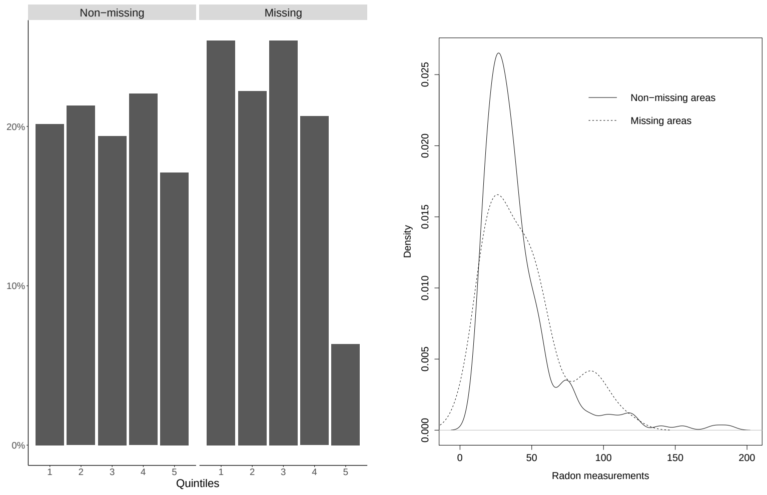

Panel A Panel B

S2 Additional material for the simulation study

S2.1 Sensitivity analysis to evaluate identifiability of the imputation model under the assumption of missing not at random (MNAR) mechanism for the generalized EPS

We carried out several sensitivity analyses to detect a possible lack of identifiability in the missingness model under MNAR assumption, as specified in (5) of the main paper (Section 3.4.2). The concern here is about the identifiability of the so called *sensitivity parameter* describing the degree of MNAR, that is the parameter a_Z, as there is no information in the data to inform it. To implement the sensitivity analyses, we followed Mason *et al.* (2012). We used the generative model described for the simulation design 3 in the main paper and we assumed a linear scenario. We set up nine analyses in which the parameter a_Z was fixed to values {-5, -2.5, -1.5, -1, 0, 1, 1.5, 2.5, 5}. Note that the analysis with $a_Z = 0$ is equivalent to assuming that the missingness mechanism is missing at random (MAR). The true value of a_Z in the simulation study was 1. We assessed how inference about the parameter of interest (β_X, fixed at 0.2 in the simulation study) varies according to the value at which the sensitivity parameter was fixed. Table S1 presents the results of such sensitivity analyses, in terms of model performance for the study's target parameter β_X (100 simulated data sets). It also provides the results of the model where a_Z is not fixed, but has a zero-centred Normal prior distribution with variance fixed at 10, which is the mildly informative prior distribution originally considered in our model (see Table 1 of the main paper). Note that the models constraining the parameter a_Z to the implausible values of {-5, 5} are not reported in Table S1 as WinBUGS crashed. This provides a piece of important information, as shows that the model does not work if contradictory evidence is forced upon it through the prior.

Table S1: Parameter estimation for β_X under the sensitivity analysis for the parameter a_Z. Posterior mean, mean bias (MB), root mean square error (RMSE), coverage and width of 95% CI. Results from 100 replicated data sets.

Value set for a_Z	Post. mean	MB	RMSE	Coverage	Width
a_Z=-2.5	0.29	0.09	0.13	87%	0.28
a_Z=-1.5	0.24	0.04	0.10	98%	0.25
a_Z=-1	0.22	0.02	0.09	100%	0.24
a_Z=0	0.22	0.02	0.08	90%	0.18
a_Z=1	0.19	-0.01	0.07	100%	0.21
a_Z=1.5	0.19	-0.01	0.07	92%	0.18
a_Z=2.5	0.19	-0.01	0.07	100%	0.22
$a_Z \sim \mathrm{N}(0, 10)$	0.20	0.00	0.07	94%	0.18

The posterior mean of the sensitivity parameter a_Z when it is estimated is 2.08 (95%CI: 0.29 to 4.38).

From the results obtained in these simulated analyses, it is apparent that the imputation methodology under MNAR assumption is reasonably robust.

Unless prior knowledge about the missingness mechanism exists, we strongly recommend the readers to perform sensitivity analyses for this parameter, exploring a wide range of potential values, which then are used to specify an informative prior.

S2.2 Checking plots for the generalized EPS

Figure S6 displays several checking plots on the generalized EPS for the simulation designs 2 (Panel A) and 3 (Panel B), respectively built up under the assumptions of MAR and MNAR mechanism. The plots present the relationships between the "true" generalized EPS obtained as the linear combination of $\mathbf{M}_i$ with coefficients specified in (6) of the main paper and the imputed generalized EPS (posterior mean) in the analysis stage. Results are from a randomly selected simulated data set (DS27).

Figure S6: Generalized EPS plots under assumption of MAR and MNAR mechanism

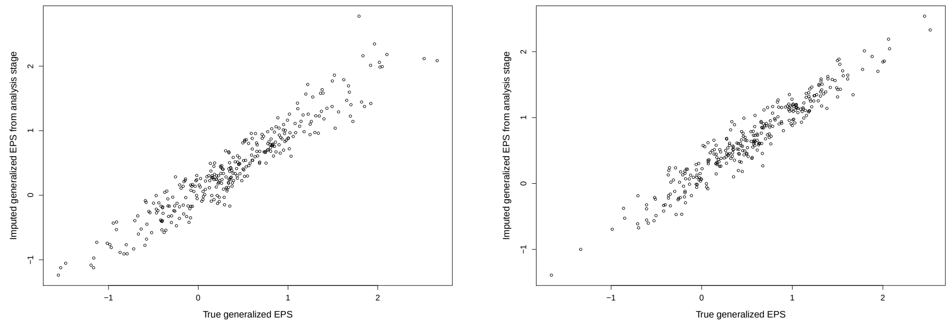

Simulation design 2 - Panel A Simulation design 3 - Panel B

S2.3 Additional simulations assuming a 30% of sparsity for the individual-level potential confounders and assuming different health effect sizes

We describe the simulation performed to evaluate the performance of the proposed methodological approach assuming that approximately the 30% of the London's wards have no information on the individual-level confounders. Two different values for the log relative risk β_X are used: 0.2 and 0.00. The first of these reflects the magnitude of the risks commonly observed in air pollution studies (it is the one used also in the main paper where we assume that the 50% of the London's wards have no information on the individual-level confounders), while the latter is chosen to evaluate the possibility of bias in the case of null risk.

We use the same simulation framework presented in the main paper, Section 4.1, but now we generate the two binary missing value indicators, $l \in \{\text{MAR}, \text{MNAR}\}$ for $i = 1, \dots, N$ such that:

$$\ell_i^l = \begin{cases} 0 & \text{if } m_{ijk} \text{ is observed} \\ 1 & \text{if } m_{ijk} \text{ is missing} \end{cases}$$

from Bernoulli distributions with probabilities defined as follows:

- MAR design: $\mathbf{P}(\ell_i^{\text{MAR}} = 1 | X_i, C_i) = \frac{\exp(-1.5+0.5X_i+0.5C_i)}{1+\exp(-1.5+0.5X_i+0.5C_i)}$,
- MNAR design: $\mathbf{P}(\ell_i^{\text{MNAR}} = 1 | X_i, C_i, Z_i^{\text{True}}) = \frac{\exp(-2+1X_i+0.5C_i+1Z_i^{\text{True}})}{1+\exp(-2+1X_i1+0.5C_i+1Z_i^{\text{True}})}$.

Here, Z_i^{True} is the linear combination of $\mathbf{M}_i$, with coefficients specified in equation (6) of the main paper.

The findings from 100 simulated data sets appear in Tables S3 and S4, which present the results when the true value of β_X is equal to 0.2 and 0.00 respectively. Note that the estimates for *simulation design 1* in Table S3 are equal to the results reported in Table 1 of the main paper, as here we

adopt the same simulating setting, but with a different proportion of missing individual-level data in space. For both the new simulative examples, we found that the results are reasonably in line with those reported in the main paper. However, because the level of spatial coverage of the individual-level potential confounders is higher than the one considered in the main paper, the bias and the uncertainty associated with the analyses seem in general less severe.

Table S2: Parameter estimation for β_X in the three simulation designs, where $\mathbf{m}_{ij}$ are assumed fully observed (design 1) and spatially sparse respectively under a MAR (design 2) and MNAR (design 3) mechanisms (target value β_X=0.20). Posterior mean (95% credible interval (CI)), mean bias (MB), root mean square error (RMSE), coverage and width of 95% CI. Note that N refers to the total number of areas, while S refers to the subset of the areas with survey data. Results from 100 replicated data sets.

Models	Post. mean (95% CI)	MB	RMSE	Coverage	Width
**Simulation design 1:**					
**All the variables are fully observed**					
Linearity between Y_i *and* $\mathbf{M}_i$					
True (benchmark) model	0.20 (0.13 to 0.27)	0.00	0.04	95%	0.11
Naïve model	0.80 (0.76 to 0.83)	0.60	0.60	0%	0.05
Z_i adj	0.21 (0.12 to 0.29)	0.01	0.04	94%	0.12
Nonlinearity between Y_i *and several* $\mathbf{M}_i$					
True (benchmark) model	0.20 (0.14 to 0.27)	0.00	0.04	95%	0.09
Naïve model	0.82 (0.70 to 0.94)	0.62	0.62	0%	0.11
Z_i adj via polynomial function	0.19 (-0.02 to 0.39)	-0.01	0.11	87%	0.26
Z_i adj via linear function	0.20 (-0.05 to 0.45)	0.00	0.13	90%	0.31
**Simulation design 2:**					
**Sparsity under MAR mechanism**					
Linearity between Y_i *and* $\mathbf{M}_i$					
Z_i adj, $i \in S$ (complete case analysis)	0.23 (0.12 to 0.42)	0.03	0.08	96%	0.18
Z_i imp & adj, $i \in N$	0.21 (0.10 to 0.39)	0.01	0.07	88%	0.13
Nonlinearity between Y_i *and several* $\mathbf{M}_i$					
Z_i adj, $i \in S$ (complete case analysis)	0.21 (-0.05 to 0.48)	0.01	0.13	80%	0.26
Z_i imp & adj via polynomial function	0.21 (-0.03 to 0.46)	0.01	0.13	78%	0.26
Z_i imp & adj via linear function	0.18 (-0.15 to 0.47)	-0.02	0.15	85%	0.36
**Simulation design 3:**					
**Sparsity under MNAR mechanism**					
Linearity between Y_i *and* $\mathbf{M}_i$					
Z_i adj, $i \in S$ (complete case analysis)	0.20 (0.09 to 0.32)	0.00	0.06	93%	0.16
Z_i imp & adj, $i \in N$	0.18 (0.08 to 0.29)	-0.02	0.06	100%	0.17
Nonlinearity between Y_i *and several* $\mathbf{M}_i$					
Z_i adj, $i \in S$ (complete case analysis)	0.33 (0.09 to 0.56)	0.13	0.18	74%	0.35
Z_i imp & adj via polynomial function	0.17 (-0.07 to 0.41)	-0.03	0.12	96%	0.36
Z_i imp & adj via linear function	-0.16 (-0.45 to 0.12)	0.36	0.39	10%	0.44

Table S3: Parameter estimation for β_X in the three simulation designs, where $\mathbf{m}_{ij}$ are assumed fully observed (design 1) and spatially sparse respectively under a MAR (design 2) and MNAR (design 3) mechanisms (target value β_X=0.00). Posterior mean (95% credible interval (CI)), mean bias (MB), root mean square error (RMSE), coverage and width of 95% CI. Note that N refers to the total number of areas, while S refers to the subset of the areas with survey data. Results from 100 replicated data sets.

Models	Post. mean (95% CI)	MB	RMSE	Coverage	Width
**Simulation design 1:**					
**All the variables are fully observed**					
Linearity between Y_i and $\mathbf{M}_i$					
True (benchmark) model	0.00 (-0.07 to 0.09)	0.00	0.04	96%	0.11
Naïve model	0.60 (0.56 to 0.63)	0.60	0.60	0%	0.05
Z_i adj	0.01 (-0.08 to 0.10)	0.01	0.04	98%	0.13
Nonlinearity between Y_i and several $\mathbf{M}_i$					
True (benchmark) model	0.00 (-0.06 to 0.07)	0.00	0.03	95%	0.09
Naïve model	0.61 (0.49 to 0.74)	0.61	0.62	0%	0.11
Z_i adj via polynomial function	-0.02 (-0.22 to 0.19)	-0.02	0.11	92%	0.27
Z_i adj via linear function	-0.01 (-0.26 to 0.24)	-0.01	0.13	90%	0.33
**Simulation design 2:**					
**Sparsity under MAR mechanism**					
Linearity between Y_i and $\mathbf{M}_i$					
Z_i adj, $i \in S$ (complete case analysis)	0.03 (-0.09 to 0.22)	0.03	0.08	95%	0.19
Z_i imp & adj, $i \in N$	0.01 (-0.10 to 0.20)	0.01	0.07	90%	0.14
Nonlinearity between Y_i and several $\mathbf{M}_i$					
Z_i adj, $i \in S$ (complete case analysis)	0.01 (-0.22 to 0.31)	0.01	0.13	84%	0.28
Z_i imp & adj via polynomial function	0.01 (-0.23 to 0.27)	0.01	0.13	81%	0.26
Z_i imp & adj via linear function	-0.01 (-0.32 to 0.29)	-0.01	0.15	91%	0.38
**Simulation design 3:**					
**Sparsity under MNAR mechanism**					
Linearity between Y_i and $\mathbf{M}_i$					
Z_i adj, $i \in S$ (complete case analysis)	0.00 (-0.12 to 0.11)	0.00	0.06	98%	0.16
Z_i imp & adj, $i \in N$	0.01 (-0.12 to 0.09)	-0.01	0.06	96%	0.15
Nonlinearity between Y_i and several $\mathbf{M}_i$					
Z_i adj, $i \in S$ (complete case analysis)	0.05 (-0.20 to 0.28)	0.05	0.13	86%	0.32
Z_i imp & adj via polynomial function	-0.01 (-0.22 to 0.20)	-0.01	0.11	92%	0.28
Z_i imp & adj via linear function	0.09 (-0.24 to 0.42)	0.09	0.19	82%	0.39

S2.4 Additional simulations using diverging parameters for generating X and Y variables

Here, we describe the simulations performed to evaluate the performance of the proposed methodological approach under diverging parameters generating the exposure $\mathbf{X}$ and the health outcome $\mathbf{Y}$.

We use the same simulation framework presented in the main paper, Section 4.1, but now we generate the exposure $\mathbf{X}$ and the health outcome $\mathbf{Y}$ from the following models:

(i) $X_i \sim \mathrm{N}(0 + 0.6C_i - 0.4M_{i1} - 0.3M_{i2} + 0.6M_{i3} + 0.3M_{i4} + 0.1M_{i5}, 0.25)$;

(ii) $Y_i \sim \mathrm{Poisson}(E_i \exp(0 + 0.5X_i + 0.3C_i - 0.2M_{i1} - 0.3M_{i2} + 0.3M_{i3} - 0.3M_{i4} - 0.1M_{i5} + \theta_i)$, where $\theta_i \sim \mathrm{N}(0, 0.05)$.

Sparsity in space of the individual-level variables is generated similarly to the simulation study presented in the main paper, assuming that approximately the 50% of the London's wards have no information on the individual-level potential confounders.

Table S4 presents the results, across 100 replications, for the three simulation designs as presented in the main paper, defined by three assumptions about the spatial coverage of the individual-level variables, that are (i) *simulation design 1* with full spatial coverage, (ii) *simulation design 2* with sparse spatial coverage under MAR mechanism, and (iii) *simulation design 3* with sparse spatial coverage under MNAR mechanism. Results are for a linear scenario, which assumes a linear dependence between the up-scaled confounding factors $\mathbf{M}_i$ and the outcome variable $\mathbf{Y}$.

Table S4: Parameter estimation for β_X in the three simulation designs, where $\mathbf{m}_{ij}$ are assumed fully observed (design 1) and spatially sparse respectively under a MAR (design 2) and MNAR (design 3) mechanisms (target value β_X=0.50). Posterior mean (95% credible interval (CI)), mean bias (MB), root mean square error (RMSE), coverage and width of 95% CI. Note that N refers to the total number of areas, while S refers to the subset of the areas with survey data. Results from 100 replicated data sets.

Models	Post. mean (95% CI)	MB	RMSE	Coverage	Width
**Simulation design 1:**					
**All the variables are fully observed**					
True (benchmark) model	0.50 (0.42 to 0.58)	0.00	0.04	96%	0.11
Naïve model	1.04 (1.00 to 1.08)	0.54	0.54	0%	0.06
Z_i adj	0.48 (0.37 to 0.58)	-0.02	0.06	95%	0.15
**Simulation design 2:**					
**Sparsity under MAR mechanism**					
Z_i adj, $i \in S$ (complete case analysis)	0.48 (0.34 to 0.65)	-0.02	0.08	88%	0.21
Z_i imp & adj, $i \in N$	0.48 (0.31 to 0.70)	-0.02	0.09	83%	0.22
**Simulation design 3:**					
**Sparsity under MNAR mechanism**					
Z_i adj, $i \in S$ (complete case analysis)	0.47 (0.29 to 0.65)	-0.03	0.09	93%	0.25
Z_i imp & adj, $i \in N$	0.47 (0.27 to 0.66)	-0.03	0.10	91%	0.26

In these additional simulative examples, we used a larger target parameter (i.e. β_X=0.5) in comparison to the one used in the simulation study presented in the main paper. In presence of a stronger signal to be estimated, we did not find relevant differences between a complete case analysis, which considers only the adjustment for the generalized EPS for the in-sample areas, and a full analysis, which includes an additional imputation model for the generalized EPS for the out-of-sample areas.

However, a so strong signal in unlikely to be observed in environmental health effect studies, where typically the target of inference is a small risk.

References

Mason, A., Richardson, S., Plewis, I., and Best, N. (2012). Strategy for modelling nonrandom missing data mechanisms in observational studies using Bayesian methods. *Journal of Official Statistics* **28**, 279–302.

Vienneau, D., de Hoogh, K., Beelen, R., Fischer, P., Hoek, G., *et al.* (2010). Comparison of land-use regression models between great Britain and the Netherlands. *Atmospheric Environment* **44**, 688–696.
